# Luminescent Lanthanide Metal Organic Frameworks as Chemosensing Platforms towards Agrochemicals and Cations

**DOI:** 10.3390/s19051260

**Published:** 2019-03-12

**Authors:** Germán E. Gomez, María dos Santos Afonso, Héctor A. Baldoni, Federico Roncaroli, Galo J. A. A. Soler-Illia

**Affiliations:** 1 Gerencia Química, Centro Atómico Constituyentes, Comisión Nacional de Energía Atómica, Avenida General Paz 1499, San Martin, Buenos Aires 1650, Argentina; 2 Instituto de Investigaciones en Tecnología Química (INTEQUI), Área de Química General e Inorgánica, Facultad de Química, Bioquímica y Farmacia, Chacabuco y Pedernera, Universidad Nacional de San Luis, San Luis 5700, Argentina; 3 Departamento de Química Inorgánica, Analítica y Química Física, Facultad de Ciencias Exactas y Naturales, Universidad de Buenos Aires, Ciudad Universitaria Pabellón II 3er Piso, Intendente Guiraldes, Ciudad Autónoma de Buenos Aires C1428EGA, Argentina; dosantos@qi.fcen.uba.ar; 4 Instituto de Química Física de los Materiales, Medio Ambiente y Energía (INQUIMAE)-CONICET, Ciudad Universitaria Pabellón II 3er Piso, Intendente Guiraldes, Ciudad Autónoma de Buenos Aires C1428EGA, Argentina; 5 Instituto de Matemática Aplicada de San Luis (IMASL), Área de Química General e Inorgánica, Facultad de Química, Bioquímica y Farmacia, Chacabuco y Pedernera, Universidad Nacional de San Luis, San Luis 5700, Argentina; hbaldoni@unsl.edu.ar; 6 Departamento de Física de Materia Condensada, Centro Atómico Constituyentes, Comisión Nacional de Energía Atómica (CNEA), Avenida General Paz 1499, San Martín, Buenos Aires 1650, Argentina; f_roncaroli@hotmail.com; 7 Instituto de Nanosistemas, Universidad Nacional de San Martín-CONICET, Av. 25 de Mayo 1021, San Martín, Buenos Aires 1650, Argentina

**Keywords:** metal organic frameworks (MOFs), sensing, luminescence, lanthanides

## Abstract

Since the first studies of luminescent sensors based on metal organic frameworks (MOFs) about ten years ago, there has been an increased interest in the development of specific sensors towards cations, anions, explosives, small molecules, solvents, etc. However, the detection of toxic compounds related to agro-industry and nuclear activity is noticeably scarce or even non-existent. In this work, we report the synthesis and characterization of luminescent lanthanide-based MOFs (Ln-MOFs) with diverse crystalline architectures obtained by solvothermal methods. The luminescent properties of the lanthanides, and the hypersensitive transitions of Eu^3+^ (^5^D_0_→^7^F_2_) and Tb^3+^ (^5^D_4_→^7^F_5_) intrinsically found in the obtained MOFs in particular, were evaluated and employed as chemical sensors for agrochemical and cationic species. The limit of detection (LOD) of Tb-PSA MOFs (**PSA** = 2-phenylsuccinate) was 2.9 ppm for [UO_2_^2+^] and 5.6 ppm for [Cu^2+^]. The variations of the 4f–4f spectral lines and the quenching/enhancement effects of the Ln-MOFs in the presence of the analytes were fully analyzed and discussed in terms of a combinatorial “host–guest” vibrational and “in-silico” interaction studies.

## 1. Introduction

In early studies, metal organic frameworks (MOFs) and coordination polymer (CP) compounds have been the center of an intensely developed field due to their emerging applications in ion exchange and gas separation [1] and heterogeneous catalysis [2,3,4,5]. In addition, the possibility of using a diversity of structural building blocks allows chemists to design these frameworks in order to exploit specific and/or tunable properties stemming from the ions, the molecular linkers, and their connectivity and synergy. In this sense, lanthanide ions are of special interest in photonics and magnetism because of their unique optical features derived from 4f–4f transitions which include narrow signals and a wide range of lifetime values, frequently accompanied with high quantum yields (QYs) [6,7,8]. These features are important in materials science for the specific design of phosphors [9], the generation and amplification of light in lasers [10], optical amplifiers [11], solid-state lighting (SSL), full color displays, and backlights [12,13,14]. Moreover, the hypersensitive transitions [15] found in some lanthanides enable Ln-MOFs and/or Ln-CPs to be suitable candidates for the elaboration of thermo- [16,17] or chemical sensors for toxic substances [18]. In particular, the ^5^D_0_→^7^F_2_ in Eu^3+^ and ^5^D_4_→^7^F_5_ in Tb^3+^ are the most reliable and robust signals for those proposals (see Supplementary Material Section 1) [19,20].

On the other hand, microporous MOFs are an attractive kind of materials that exhibit fascinating environment-responsive properties, such as “breathing” and “swelling”, which are involved in selective sorption and controlled release of molecules from MOFs used in sensing and drug delivery, respectively [21]. Given the richness of terminal functional groups of the linkers and solvent molecules within MOFs to induce H-bonding interactions with guest species, open frameworks are expected to be promising materials for chemical sensing. There are already remarkable examples of 3D, open structures such as Ln-BTC (BTC = 1,3,5-benzenetricarboxilate) acting as cationic [22] or small molecule [23] sensors. Moreover, the set of Ln-BPDC phases (Ln^3+^ = Tb, Ho, Er and Y; BPDC = 4,4′-biphenyldicarboxylate) presents one-dimensional micropores which are suitable for sensing assays [24].

Contrary to open frameworks such as zeolites and MOF materials, some structures, such as 3D or 2D CPs, cannot exhibit the “confinement effect” [25]. Nevertheless, their optical properties could be useful for sensing due to the hypersensitive nature of the luminescence of their components. Three examples of dense lanthanide-based frameworks with sensing applications obtained in our group are the 3D europium carboxylate which has been used to sense small solvents; [26] meanwhile, the 2D mixed compound, EuTb-PSA (PSA = 2-phenylsuccinate), was employed as a thermometric sensor in the 13.5–313.5 K range [27]. In addition, a 1D framework was recently obtained by us and studied as a chemical sensor towards Volatile Organic Compounds (VOCs) with excellent results [28]. Moreover, a new 2D mixed phase doped with Eu and Tb was synthesized and tested as a chemosensor for small molecules showing selectivity towards carbonyl compounds [29].

As it is necessary to design new methodologies to detect toxic and dangerous compounds in an accurate way, the use of luminescent MOFs (LMOFs) seems to be a reliable pathway to non-conventional sensor platforms [18]. In the recent literature, there are many examples of LMOFs as sensors of explosives, cations, anions, solvents, and VOCs, among others [30], but strikingly, there have been no reports for agrochemical sensing. This is indeed a strategic field due to the need for more efficient methods of mass food production. In addition, periodic or prolonged contact with agrochemicals has been documented to lead to negative effects on human health, such as neuronal illnesses (fungicides, insecticides, and fumigants), cancer, immunologic abnormalities, and reproductive damage (pesticides) [31,32].

Given the nearly limitless choices of building block combinations, MOFs thrive on structural diversity and tunable chemical and physical exceptional properties. The permanent porosity in a large set of MOFs further enables the adsorption of guest molecules, enhancing host–guest interactions. The pore size, shape, chemical composition, surface, and molecular environment can be finely tuned, potentially facilitating the selective seizing of certain guest molecules. This merit of MOFs is the foundation of many well-explored applications, especially in gas storage and separation fields. In addition, the perturbation from adsorbed guest molecules can alter the photoluminescence in LMOFs, making great candidates for chemosensing.

For LMOFs, in principle, any change in their spectroscopic characteristics can potentially be used as a sensing signal. While the most commonly used feature is the fluorescence intensity, depending on the electronic nature of the molecule being detected (analyte), either quenching or enhancement of the luminescence can be exploited. These changes can be attributed to either electron transfer or energy transfer (ET) between the analyte species and the LMOF, or a combination of both [33,34,35,36]. Nitroaromatic compounds, such as explosive-like molecules, are known as strong quenchers due to their high electron affinity [37]. In addition, paramagnetic metal ions (i.e., Cu^2+^, Mn^2+^, Ni^2+^, Co^2+^) are also capable of quenching fluorescence since they can facilitate ligand to metal charge transfer (LMCT) and relax the excitation energy through non-radiative pathways [38,39]. In contrast, electron-rich molecules such as benzene and its derivatives with electron-donating substituents, can efficiently enhance fluorescence due to their ability to donate an electron from an excited state to the LUMO (conduction band) of the LMOF [40,41]. 

In this work we present an in-depth study of the sensor activity of two 3D LMOFs, **Eu-BTC** and **Eu-BPDC**, and two layered CPs, **Eu-PSA** and **Tb-PSA**, obtained by solvothermal method. All compounds were fully characterized by X-ray powder diffraction (PXRD), vibrational spectroscopy (FTIR), thermal analysis (TGA-DSC), and scanning electron microscopy (SEM). Regarding the optical features of these materials, their chemical sensor activities were evaluated on the basis of the influence of diverse agrochemicals and cationic species on the lanthanides hypersensitive ^5^D_0_→^7^F_2_ and ^5^D_4_→^7^F_5_ signals. All these studies mark the route for the rational design of luminescent Ln-MOFs and set the basis for building specific optical devices based on pre-designed components with synergetic, adsorptive-luminescent functionality.

## 2. Materials and Methods

Caution: Whereas the uranium nitrate (UO_2_(NO_3_)_2_ × 6H_2_O) used in this study consists of depleted U, standard precautions for handling radioactive and toxic substances should be followed. The same guidelines were carried out when agro-toxics were used. 

### 2.1. Synthesis of Sensor Materials

The sensor materials were obtained as crystalline solids under solvothermal conditions using 43 or 120 mL Teflon-lined Parr reactors. Crystalline samples of [Eu_2_(C_10_H_8_O_4_)_3_(H_2_O)] (**Eu-PSA** or **Tb-PSA**, [Eu_2_(C_14_H_8_O_4_)_3_(H_2_O)_2_]. DMF (**Eu-BPDC**) and [Eu_2_(C_9_H_3_O_4_)_3_(H_2_O)] × DMF (**Eu-BTC**) were prepared according to procedures similar to those previously reported [24,42,43]. The detailed synthetic procedure is presented in Supplementary Material Section 2).

### 2.2. Powder X-Ray Diffraction (PXRD) 

X-ray powder diagrams were obtained with an Empyrean difractometer equipped with a Pixcel 3D detector, CuKα radiation source (λ = 1.5418 Å) 40 mA, 40 kV. The best counting statistics were achieved using a scanning step of 0.05° between 5° and 40° Bragg angles with an exposure time of 5 s per step. The PXRD of all powdered samples were compared with the respective simulated patterns and showed in Supplementary Material Section 3. The structure conservation of the sensors after thermal treatment and toxics expositions were also analyzed by PXRD (see Supplementary Material Section 4).

### 2.3. Thermal Analysis 

Thermogravimetric analysis (TGA) and differential thermal analysis (DTA) were simultaneously performed with Shimadzu TGA-51, DTA-50 apparatus (Shimadzu, Japan) under flowing air at a flow rate of 50 mL min^−1^ and a heating rate of 10 °C min^−1^. Vibrational spectroscopy and thermal analysis were carried out in order to check the reported formula (Supplementary Material Section 5).

### 2.4. Fourier Transform Infrared Spectroscopy (FTIR) 

FTIR spectra were recorded with a Nicolet Protégé 460 spectrometer in the range 4000–600 cm^−1^; spectra were recorded averaging 64 scans and with a spectral resolution of 4 cm^−1^ by the KBr pellet technique. 

### 2.5. Scanning Electron Microscopy (SEM) and Energy-Dispersive X-ray Spectra (EDS)

Crystal morphology and EDS semiquantitative analysis were performed on an FEI Quanta 400 microscope. 

### 2.6. UV Absorption Spectroscopy

UV Absorption Spectra of Analytes were Recorded with a UV Spectrophotometer-160A (Shimadzu, Japan).

### 2.7. Luminescence Measurements

For sensing measurements, suspensions containing 1.5 mg of the activated MOF (for **Eu-BPDC** and **Eu-BTC**) in 3 mL of the methanolic solutions were ultrasonicated for 30 min in a Clausius apparatus for an efficient analyte-sensor contact. For sensor activation procedures, see Supplementary Material Sections 4–6. The measurements were carried out in a Félix X32 PTI spectrofluorometer with Xe UXL-75 Xe lamp employing a quartz cell of 1 cm of optical path (Horiba, Japan). The chosen excitation wavelengths were 393 nm and 352 nm for Eu-MOFs and Tb-MOFs, respectively. The monitored 4f–4f hypersensitive transitions for Eu^3+^ and Tb^3+^ were ^5^D_0_→^7^F_2_ (615 nm) and ^5^D_4_→^7^F_5_ (541 nm). 

### 2.8. Simulation Details

System setup: Classical MD simulations reported in this paper were performed using the Amber16 program suite [44]. The all-atoms system was represented by the general AMBER force field (GAFF) set of parameters, while the europium parameters were published elsewhere [45,46,47]. Partial atomic charges were calculated by the AM1BCC method [48,49] using the antechamber set of auxiliary programs [44].

The atomistic description of the host frameworks was constructed from the experimental crystallographic data taken from previous studies [45,46,47]. A supercell representation having 3 × 3 × 3 primitive cells was sufficient in our case to allow us to embed the guest molecules and obtain reliable results. The supercell representations account for ~100Å, ~60Å, and ~58Å in the X, Y, and Z directions, respectively.

The molecular structure of the guest molecules, namely metsulfuron (**MSM**; IUPAC: 2-{[(4-methoxy-6-methyl-1,3,5-triazin-2-yl)amino]-oxomethyl]sulfamoyl}benzoic acid methyl ester), imazalil (**IMZ**; IUPAC: 1-{2-(2,4-dichlorophenyl)-2-[(prop-2-en-1-yl)oxy]ethyl}-1H-imidazole), chlorimuron (**CLM**; IUPAC: ethyl 2-[(4-chloro-6-methoxypyrimidin-2-yl)carbamoylsulfamoyl]benzoate), and chlorpyrifos (**CLP**; IUPAC: O,O-Diethyl O-3,5,6-trichloropyridin-2-yl phosphorothioate), were built from coordinates published elsewhere [50,51,52,53]. 

**Figure 1:**
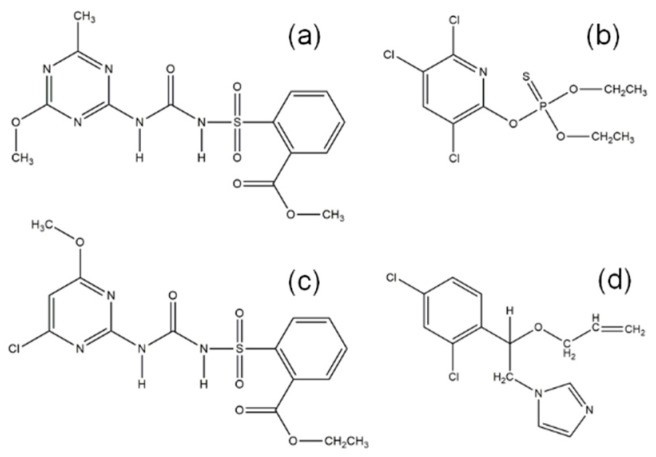
Molecular representation of the pesticides and herbicides used in this work: **MSM** (metsulfuron) (**a**), **CLP** (chlorpyrifos) (**b**), **CLM** (chlorimuron) (**c**) **IMZ** (imazalil) (**d**).

A molecular representation of the pesticides is displayed in Figure 1. Four pores within the host framework were used to confine a single representation of each guest molecule in the inside of the pore. Afterwards, the whole system was immersed in a cubic methanol box with 15 Å buffer regions. The final model system (i.e., guests: host framework:methanol box) contained ~16,962 total atoms and an approximated dimension of ~166 Å, ~127 Å, and ~127 Å in the X, Y, and Z directions respectively. The model system had its coordinates optimized and energy minimized for 10,000 steps of the steepest descendent and then equilibrated at 298 K and 1 atm in the NPT (constant number of particles (N), pressure (P), and temperature (T); T is regulated via a thermostat, which typically adds a degree of freedom to the conserved Hamiltonian) ensemble holding of the host framework as a rigid body. For a constant temperature and pressure regulation, the Berendsen thermostat and barostat were used [54]. Finally, 60 ns of production run were performed in the NVT (constant number of particles (N), volume (V), and temperature (T); T is regulated via a thermostat, which typically adds a degree of freedom to the conserved Hamiltonian) ensemble. A time step of 4 fs was used for all the simulations due to the hydrogen mass repartitioning [55] the whole model system. The particle mesh Ewald (PME) algorithm [56] together with the periodic boundary condition (PBC) was employed throughout the simulations with a 8 Å non-bonded cut-off. Snapshots were saved every 5 ps, which produced 12,000 snapshots in total for each guest molecule.

Post-processing: The saved snapshots were postprocessed to quantify the Eu:X (X = N,O,S) contacts by using the AmberTools set of auxiliary programs [44]. The stability of the contact is reflected by the fraction of contact preserved over the analyzed set of snapshots. The most stable contacts will tend to be formed within the selected cutoff of 3 Å during most of the analyzed set of snapshots, and they will show during a bigger fraction of time.

## 3. Results and Discussion

### 3.1. Synthesis of Luminescent MOFs Sensors

**Figure 2:**
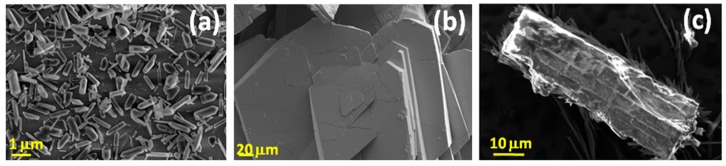
SEM micrographs of **Eu-BTC** (**a**) and **Eu-PSA** (**b**) crystalline materials. **Eu-BTC** “block” crystal after **MSM** exposition (**c**).

The described synthetic procedures in Supplementary Material Section 2 led to crystalline products (Figure 2), which were fully characterized by PXRD, TGA-DSC, and FTIR.

**Figure 3:**
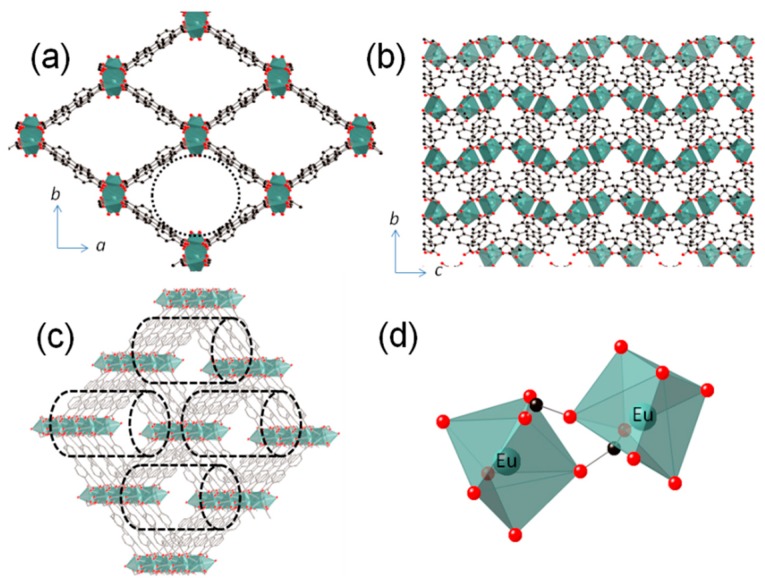
Projection on the ab and bc planes of **Eu-BPDC** (**a**,**b**), and the view of the nanochannels in the structure (**c**). The lanthanide environment of two adjacent metallic centers (**d**). Hydrogen atoms were omitted for clarity.

**Eu-BPDC** corresponds to a 3D structure compound which crystallizes into the monoclinic C2/c space group, where the asymmetric unit contains one seven-coordinated europium ion, one-and-a-half BPDC ligands, and one coordinated water molecule (see Figure 3). Each lanthanide atom is bridged by four BPDC groups, giving rise to paddle-wheel building blocks. The paddle-wheel building blocks connect each other through two carboxyl groups along the [0 0 1] direction, leading to a one-dimensional (1D) inorganic chain. These chains are linked through biphenyl groups forming the 3D framework. According to the calculations of cavity volume, the 3D framework contains 25.2 Å × 17.1 Å nanochannels along the diagonals (see Figure 3). 

**Figure 4:**
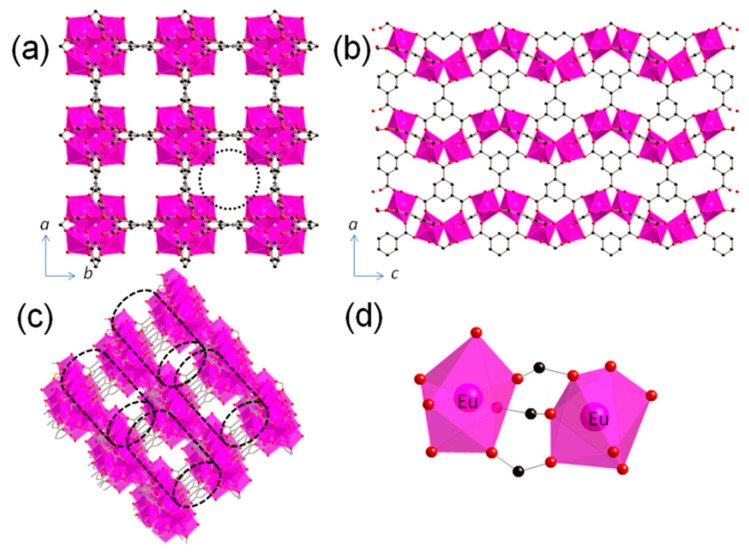
Projection on the ab and ac planes of **Eu-BTC** (**a**,**b**), and the view of the nanochannels in the structure (**c**). The lanthanide environment of two adjacent metallic centers (**d**). Hydrogen atoms were omitted for clarity.

For the case of **Eu-BTC**, the structure is 3D belonging to tetragonal P4_3_22 space group. Each asymmetric unit is composed of one seven-coordinated europium ion, one BTC linker, and one water molecule. Each lanthanide ion is surrounded by six oxygen atoms from six carboxylate groups of BTC and one oxygen atom from a terminal water molecule. The metallic chain polyhedra are developed in a helical fashion along the c axis, chains which are linked by BTC ligands along the a and b axis, giving rise to a 3D framework (see Figure 4). The structure contains unidimensional channels along the c direction, with a circular area of 36 Å^2^. Both **Eu-BPDC** and **Eu-BTC** can be described as polyhedra chains connected by ligands in two directions, conforming to a 3D framework. 

**Figure 5:**
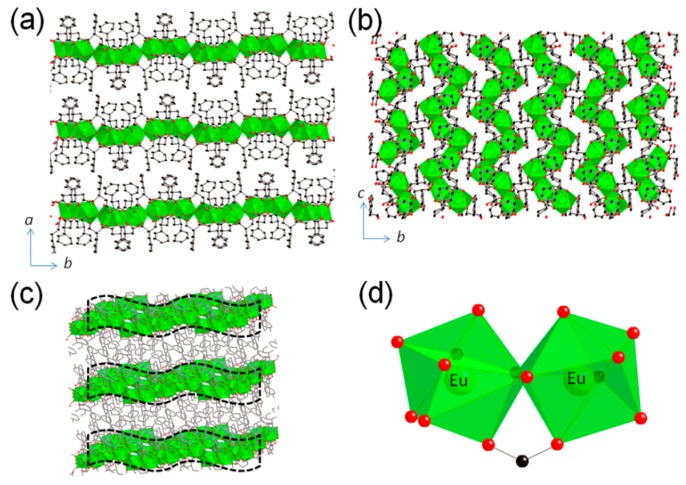
Projection on the *ab* and *bc* planes of **Eu-PSA** (**a**,**b**), and the development of the layered structure (**c**). The lanthanide environment of two adjacent metallic centers (**d**). Hydrogen atoms were omitted for clarity.

Finally, **Eu-PSA** can be described as a layered structure (monoclinic P2_1_/c space group) where the asymmetric unit is composed by two crystallographic independent europium ions with coordination numbers of 8 and 9 respectively. As can be seen in Figure 5, the framework can be described as polyhedra chains along the c direction connected by PSA ligands. As it was previously analyzed [42], no free voids were found in this structure, and this fact was a sufficient reason to classify this compound as a CP and not as MOF.

### 3.2. Sensing Essays

**Figure 6:**
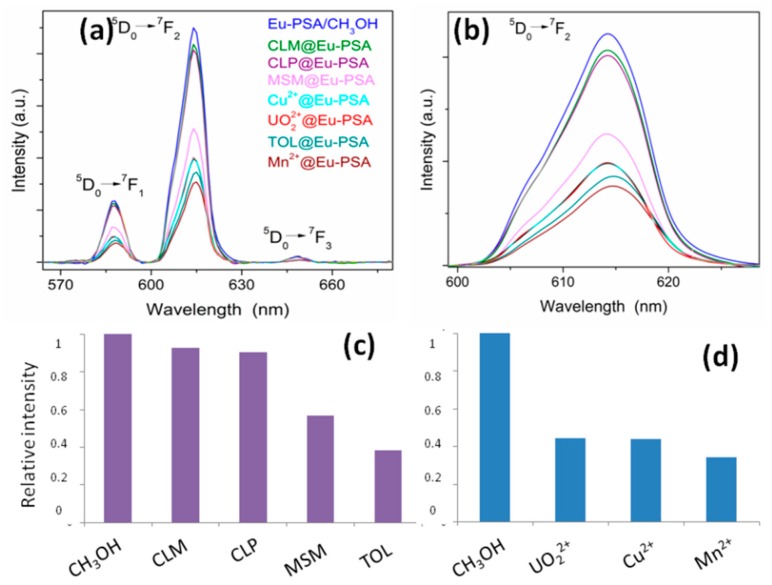
(**a**) Emission spectra of **Eu-PSA** under diverse pollutant exposition (concentration = 2 × 10^−3^ M). (**b**) Zoomed region of the Eu^3+^ hypersensitive ^5^D_0_→^7^F_2_ transition. (**c**) Relative intensities of the hypersensitive signal of **Eu-PSA** (down) in the presence of organic and (**d**) inorganic pollutants.

**Figure 7:**
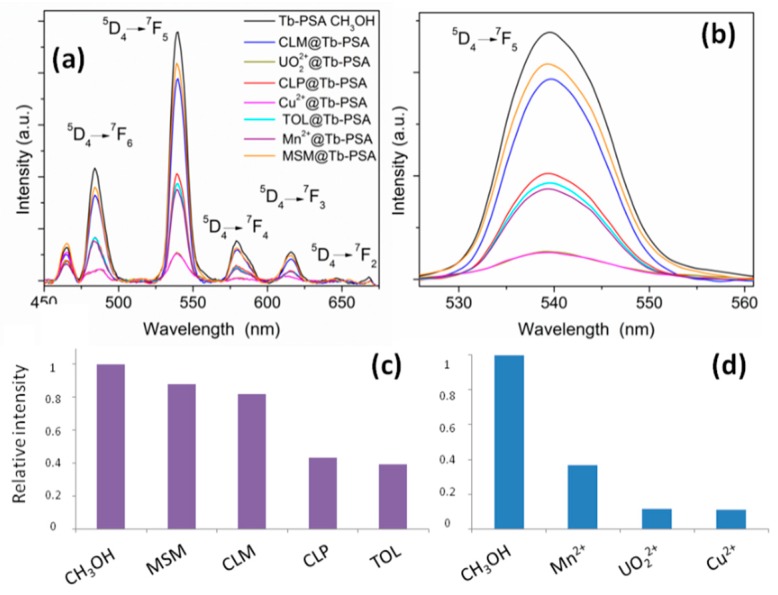
(**a**)Emission spectra of **Tb-PSA** under exposition to different pollutants (concentration = 2 × 10^−3^ M). (**b**) Zoomed region of the Tb^3+^ hypersensitive ^5^D_4_→^7^F_5_ transition. Relative intensities of the hypersensitive signal of **Tb-PSA** in the presence of organic (**c**) and inorganic (**d**) pollutants.

The optically active phases **Eu-PSA**, **Tb-PSA**, **Eu-BTC**, and **Eu-BPDC** where selected as chemosensing platforms. According to Figure 6; Figure 7, in the 2D **Eu-PSA** and **Tb-PSA** compounds, the electroaffinity of transition metal ions such as Cu^2+^, Mn^2+^, and UO_2_^2+^ justified the attenuation of quenching luminescence [38,39]. On the other hand, the quenching of 4f transitions through the organic species can be explained by electroaffinity behavior exhibited by the analytes.

**Figure 8:**
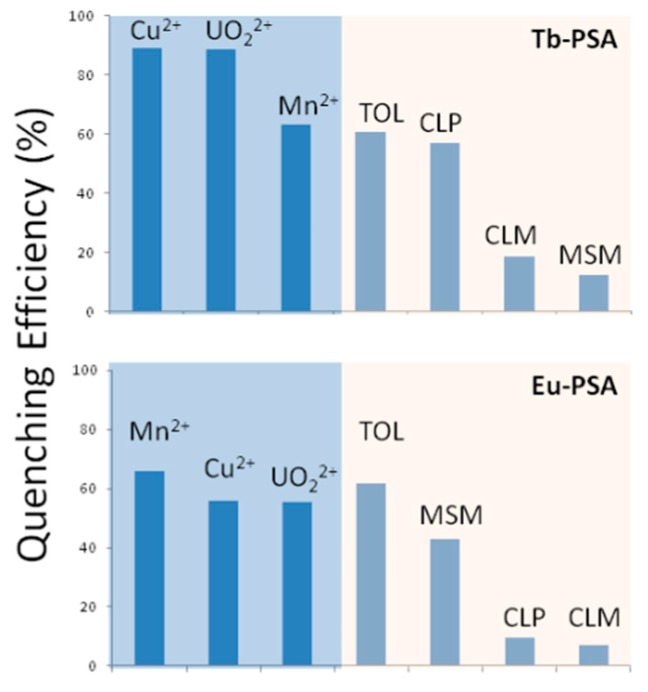
QE% of compounds **Tb-PSA** (above) and **Eu-PSA** (below) in the presence of different pollutants.

The quenching efficiency (QE) can be defined as: QE% = (I_0_ − I)/I_0_ × 100, where I_0_ and I represent the emission intensity values in the absence and in the presence of the analytes at the same concentration (2 × 10^−3^ M), respectively. Based on these results, Cu^2+^ and [UO_2_]^2+^ cationic species represent efficient quenchers for the luminescence of **Tb-PSA**, exhibiting a QE of 89%. Regarding the organic molecules, CLP exhibits a QE% of 60%, a value comparable to that of toluene (TOL) (Figure 8). As shown in Supplementary Material Section 8, these analytes show UV absorption bands ranging from 200 to 350 nm. The absorption peak of the ligand within **Eu-PSA** (or **Tb-PSA**) is overlapped by the absorbing range of the toxics, which implies that a competition of the excitation energy between the organic linker (PSA) and the analytes takes place. The majority of the toxics absorb most of the energy, and only a small fraction of energy will be transferred through the ligand to the Tb^3+^ or Eu^3+^ ions. This also illustrates why other pollutant molecules present similar quenching effects on the luminescence intensity of **Tb-PSA** and **Eu-PSA**.

**Figure 9:**
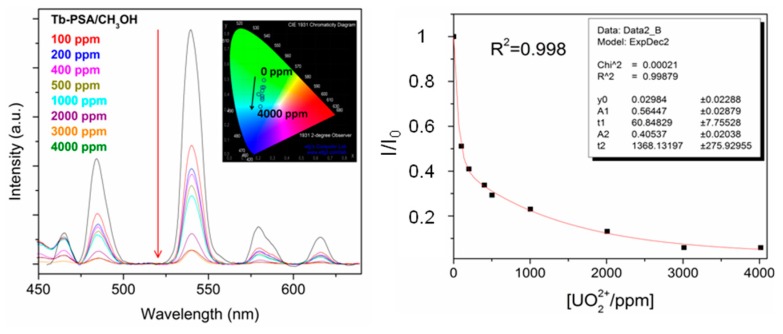
Left: Emission spectra and Comission Internationale de l´Éclairage (CIE x,y) chromaticities of **Tb-PSA** from 0 to 4000 ppm of [UO_2_]^2+^ (inset). Right: Relative intensity (I/I_0_) of the Tb^3+^ hypersensitive transition.

**Figure 10:**
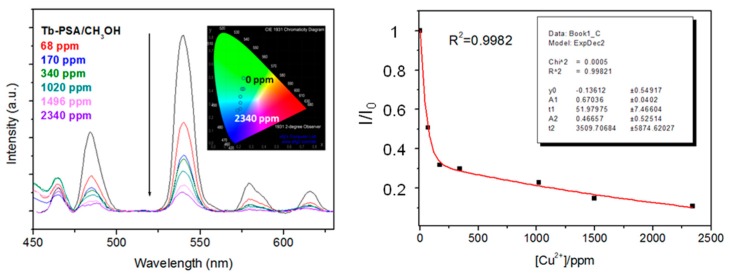
Left: Emission spectra and CIE x,y chromaticities of **Tb-PSA** from 0 to 2340 ppm of Cu^2+^. Right: Relative intensity (I/I_0_) of the Tb^3+^ hypersensitive transition.

With the purpose of evaluating the inorganic pollutant concentration effect on the PL intensity of **Tb-PSA**, two cationic species of environmental relevance and high QE were chosen (Cu^2+^ and [UO_2_]^2+^). Figure 9 shows that a gradual increase in the uranyl concentration produced a decrease in the ^5^D_4_→^7^F_5_ hypersensitive signal with an intensity quenching of 94% at a maximum [UO_2_]^2+^ concentration of 4000 ppm. The relative intensity decreases markedly with the uranyl concentration, following a biexponential behavior. This suggests that uranyl species can intervene in at least two main radiative transition paths after excitation. In a first approximation, the effect of uranyl in the fluorescence quenching processes was modeled according to the Stern–Volmer law, [57] I_0_/I = 1 + K_SV_ × [UO_2_]^2+^. In this equation, I_0_ and I are the luminescent intensities of the ^5^D_4_→^7^F_5_ transition of **Tb-PSA** before and after being exposed to different concentrations of [UO_2_]^2+^ respectively, K_SV_ being the quenching coefficient (see the graphics in Supplementary Material Sections 9 and 10). An equivalent study for Cu^2+^ ions in the range 0–2340 ppm leads to similar responses (see Figure 10). In both cases, the correlation obtained in the quenching effect is not completely linear. This effect is more marked at higher copper(II) or uranyl concentrations, reflecting multiple interactions between the ions and the MOF matrices. We can speculate that the observed biexponential intensity decay with increasing metal concentration can reflect multiple ion–MOF interactions, such as adsorption in different surface sites or remote energy transfer. However, in the lower concentration range, the agreement with the Stern–Volmer model is far better, supporting that a quenching process of diffusional nature takes place in dilute solutions. These observations support the fact that the bidimensional architectures **Tb-PSA** and **Eu-PSA** are closed to guest analytes; therefore, only superficial dynamic quenching can take place in these compounds. In addition, these experiments demonstrate the potential of detecting cationic species by quenching at low concentrations. The limit of detection (LOD) of the proposed method employing **Tb-PSA** was calculated to be 2.9 ppm for [UO_2_^2+^] and 5.6 ppm for [Cu^2+^]. Note that LOD is defined by the equation LOD = 3S_0_/K, where S_0_ is the standard deviation of blank measurements (n = 10) and K is the slope of the calibration curve. An in-depth analysis is in progress in order to compare the MOF architectures with respect to analytical performance in parameters such as sensitivity and selectivity, which will be reported in a forthcoming paper.

**Figure 11:**
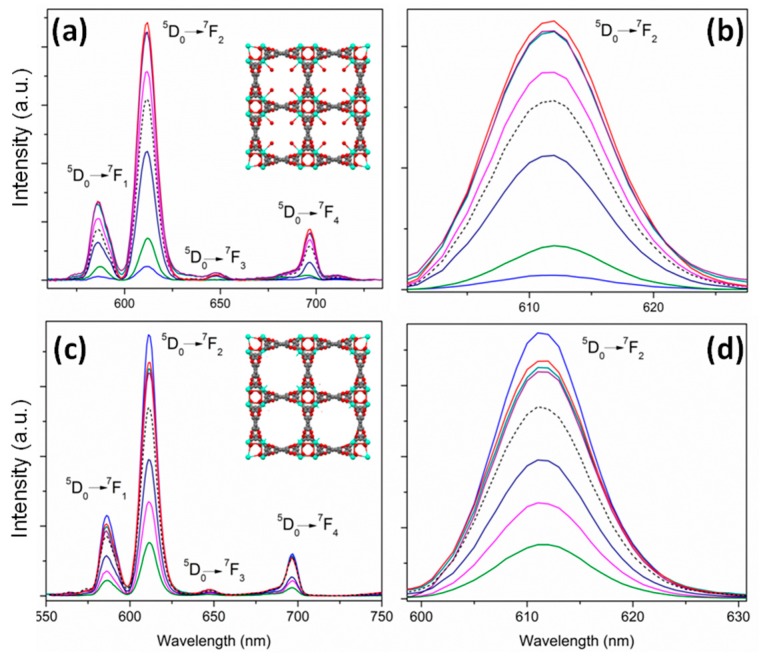
Emission spectra of **X@Eu-BTC** (**a**) and **X@Eu-BTC’** (**c**) under diverse pollutant exposition (blue: **MSM**; purple: **CLM**; pink: **CLP**; red: **TOL**; dark yellow: **IMZ**; olive: UO_2_^2+;^; deep-blue: Cu^2+^) (**a**). Black dashed line spectra correspond to those of **Eu-BTC** and **Eu-BTC’** in methanol without analyte species. Zoomed region of the Eu^3+^ hypersensitive ^5^D_0_→^7^F_2_ transition (**b**,**d**).

**Figure 12:**
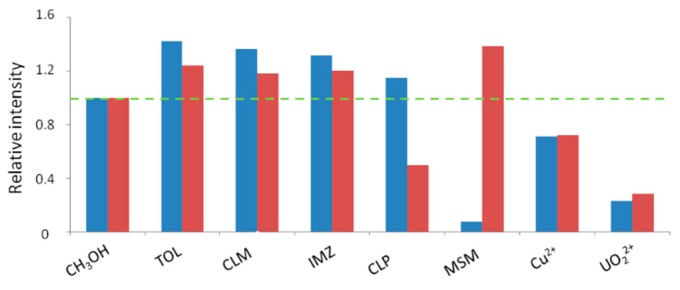
^5^D_0_→^7^F_2_ intensities of **X@Eu-BTC** (blue) and **X@Eu-BTC’** (red). The dashed green line denotes the systems without analyte presence.

In the case of 3D porous MOFs, thermal activation was made prior to the sensing studies in order to release DMF molecules from the pores, thus freeing the cavities for sensing essays. In order to study the influence of the thermal activation on the sensing performance, the luminescent properties of **Eu-BTC** and the corresponding activated material (**Eu-BTC’**) were evaluated. The BET surface area exhibited for **Eu-BTC’** (458 m^2^∙g^−1^) confirmed its potential properties as molecule–guest material. The corresponding nitrogen isotherm curve for **Eu-BTC’** (measured at 77 K) is shown in Supplementary Material Section 11. According to the results, **Eu-BTC** exhibited a marked quenching when it was contacted with MSM and cationic species as it was seen in 2D frameworks (Figure 11). Nevertheless, an intensity enhancement is identified when **Eu-BTC** is in contact with TOL, CLP, CLM, and IMZ. The differential optical behavior of **Eu-BTC** makes the MOF a potential specific sensor of toxic pollutants. We can hypothesize that the increase in the intensity is due to the energetic transfer from the analytes to the lanthanide ions provided by the “confinement effect” of the porous sensor. In addition, the thermal activation significantly improved the sensitivity, which is reflected when the ^5^D_0_→^7^F_2_ intensities of activated and nonactivated **Eu-BTC** are compared (Figure 12). Moreover, QE% of **Eu-BTC’** in the presence of CLP, [UO_2_]^2+^, and Cu^2+^ was determined as 50, 72, and 27% respectively, demonstrating a sensitization of Eu^3+^ towards the uranyl cations. In this sense, when MSM interacted with the activated material, the hypersensitive signal was sixteen times more intense than the one observed on the nonactivated compound. It seems that MSM acts as a quencher in the solvent-containing phase, but an improved analyte–matrix interaction produces an extra enhancement in the corresponding transition.

**Figure 13:**
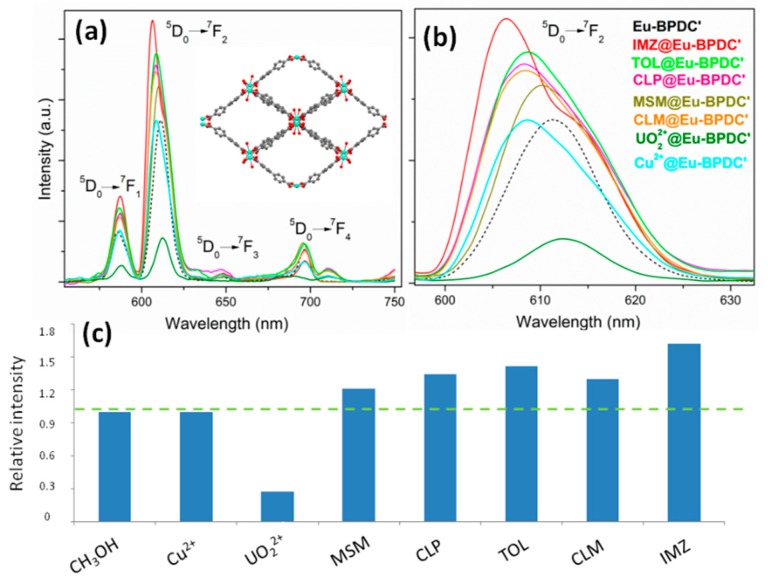
(**a**) Emission spectra of **X@Eu-BPDC’** exposed to diverse pollutants (X). (**b**) Zoomed region of the Eu^3+^ hypersensitive ^5^D_0_→^7^F_2_ transition. (**c**) Relative intensities of the hypersensitive signal of **Eu-BPDC’** in the presence of diverse pollutants, of **X@Eu-BPDC’**; the dashed green line denotes the system without analyte presence.

In the same vein, a more intimate interaction between **Eu-BPDC’** and the analytes is observed, where the hypersensitive transition suffered remarkable intensity and shape band changes mainly in ^5^D_0_→^7^F_2_ and ^5^D_0_→^7^F_4_ transitions (Figure 13a,b). In comparison with the other open framework **Eu-BTC’**, the same tendency was observed when the **Eu-BPDC’** sensor was contacted with uranyl, showing a quenching of 78% (Figure 13c).

### 3.3. Sensor–Analyte Interaction Studies

**Figure 14:**
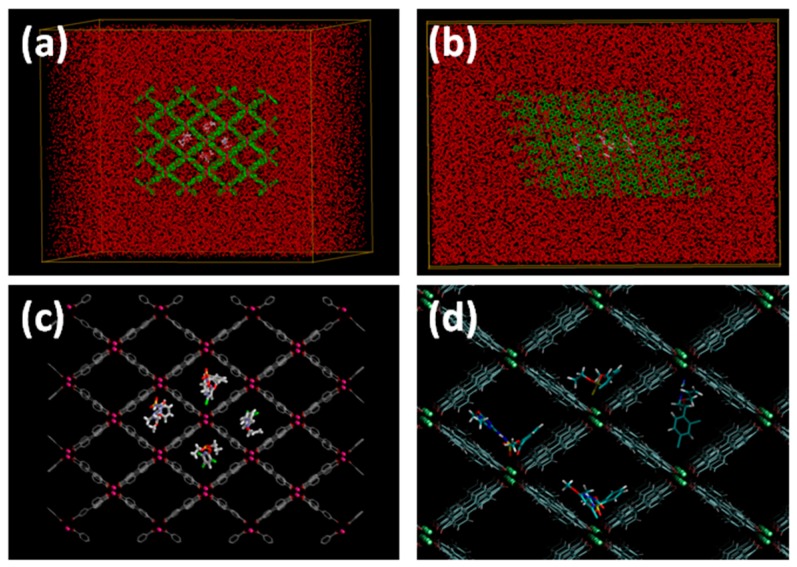
Model system of host–guest interaction (**a**,**b**) with supercell dimensions of 166 Å × 127 Å × 127 Å (total atoms number: 16,962) constructed by Amber program. Zoom of the pesticides and herbicides into the metal organic frameworks (MOF) (**c**,**d**) (MOF dimensions (x,y,z): 100 Å × 60 Å × 58 Å).

In the previous sections, a variety of optical responses was observed, which depended on the pore structure and type of interactions with the MOF matrix. In order to gain a more complete description of sensor–analyte interactions, simulations were carried out. The vibrational analysis was focused particularly on the porous **Eu-BPDC** and **Eu-BTC**, since the process of thermal activation led to stable frameworks with permanent porosity suitable for host–guest interactions assays. As stated above, no activation was necessary for **Eu-PSA** due to its layered structure, with no free voids for host–guest interactions. In addition, the luminescence studies showed that the layered phases exhibited a diffusional quenching, but a more complex and intimate interaction of the analytes took place with the porous frameworks. For this in-depth study of the contact efficiency of **X@Eu-MOF** systems involving vibrational analysis, the sensors **Eu-BTC** and **Eu-BPDC** were chosen for simplicity. A more detailed description can be found in Supplementary Material Section 12. The model system of host–guest interaction constructed employing the Amber software is depicted in Figure 14.

**Scheme 1:**
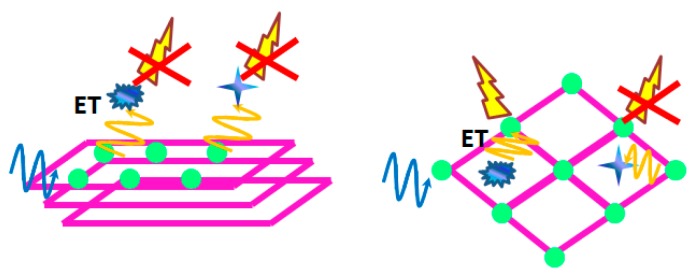
Diverse mechanisms proposed to take place in 2D **Ln-PSA** and 3D **Eu-BPDC** and **Eu-BTC**.

In light of all the results, we can state that the different sensing behavior observed is mainly due to the structure framework topology, making possible the quenching of luminescence through energy transfers between the lanthanide centers and the analytes (pesticides and cations) in the layered compounds. In addition, for open frameworks, a “confinement effect” was anticipated and a “host–guest” interaction was confirmed by a combinatorial vibrational-theoretical approach. For this reason, a mechanism involving energy transfers with quenching and enhancements of luminescent signals can be proposed in principle. Both mechanisms are outlined in Scheme 1.

## 4. Conclusions

A set of luminescent MOFs (LMOFs) based on lanthanides have been hydrothermally obtained, fully characterized, and used as potential chemosensors towards pesticides and cations. The hypersensitive transitions of ^5^D_0_→^7^F_2_ Eu^3+^ and ^5^D_4_→^7^F_5_ Tb^3+^ ions were employed to detect analytes by analyzing variations of the fluorescence intensity ratio (I/I_0_). In the case of layered coordination polymer frameworks (**Ln-PSA**), a quenching of luminescence was noticeable in the presence of cations and pesticides, which can be explained by a diffusional fluorescence quenching. This phenomenon was successfully understood in terms of the Stern–Volmer law. On the other hand, the open frameworks (**Eu-BTC** and **Eu-BPDC**) exhibited preferential quenching in the presence of paramagnetic cations, while an interaction with aromatic pesticides contributes to an enhancement in the fluorescent signal due to their rich electron nature. It is important to remark that these results are the first reports of uranyl species and pesticide sensing using MOF platforms. 

Finally, the host–guest interaction of the open frameworks was studied in terms of vibrational analysis. The dynamic simulations support the hypothesis of the importance of the interactions and confinement between the sensor and the pesticides. These results open up the potential applications of MOFs for sensing toxins of environmental interest, as well as the development of optical devices for analytical chemistry.

## Supplementary Materials

The following are available online at https://www.mdpi.com/1424-8220/19/5/1260/s1.
