# Supplementary material for: Luminescent Lanthanide Metal Organic Frameworks as Chemosensing Platforms towards Agrochemicals and Cations
Source: Sensors (Basel). 2019 Mar 12;19(5):1260. doi: 10.3390/s19051260 (PMC6427543; doi:10.3390/s19051260)

Luminescent Lanthanide Metal Organic Frameworks as chemosensing platforms towards agrochemicals and cations

G. E. Gomez*[a,b], M. dos Santos Afonso[c], H. A. Baldoni[d], F. Roncarolli[e], G. J. A. A. Soler-Illia*[f]

a. Gerencia de Investigación y Aplicaciones, Centro Atómico Constituyentes, Comisión Nacional de Energía Atómica, Grupo de Nanoquímica. Avenida General Paz 1499, San Martin, Buenos Aires, Argentina. gegomez@unsl.edu.ar

b. Instituto de Investigaciones en Tecnología Química (INTEQUI). Área de Química General e Inorgánica "Dr. G. F. Puelles", Facultad de Química, Bioquímica y Farmacia, Chacabuco y Pedernera, Universidad Nacional de San Luis, 5700 San Luis, Argentina.

c. CONICET-Universidad de Buenos Aires, Instituto de Química Física de los Materiales, Medio Ambiente y Energía (INQUIMAE), y Universidad de Buenos Aires, Facultad de Ciencias Exactas y Naturales, Departamento de Química Inorgánica, Analítica y Química Física, Ciudad Universitaria Pabellón II 3er Piso, Intendente Guiraldes , C1428EGA Ciudad Autónoma de Buenos Aires, Argentina.

d. Instituto de Matemática Aplicada de San Luis (IMASL). Área de Química General e Inorgánica "Dr. G. F. Puelles", Facultad de Química, Bioquímica y Farmacia, Chacabuco y Pedernera, Universidad Nacional de San Luis, 5700 San Luis, Argentina.

e. Departamento de Física de Materia Condensada, Centro Atómico Constituyentes, Comisión Nacional de Energía Atómica (CNEA), Avenida General Paz 1499, San Martín, Buenos Aires, Argentina.

f. Instituto de Nanosistemas. Universidad Nacional de San Martín. Av. 25 de Mayo 1021, San Martín, Buenos Aires, Argentina.

Supplementary Material

Section 1: Energy scheme of the diverse electronic excited levels for trivalent lanthanides. The marked square denotes the hypersensitive transitions for Eu^{3+} and Tb^{3+}.

Section 2: Synthesis procedures.

Section 3: PXRD patterns of **Eu-BPDC**, **Eu-BTC**, **Tb-PSA** and **Eu-PSA** in comparison with the corresponding simulated diffractograms.

Section 4: PXRD patterns of **Eu-BPDC**, **Eu-BTC**, **Tb-PSA** and **Eu-PSA** after exposition to uranyl cation.

Section 5: TGA/DTA curves for **Eu-BPDC** (left) and **Eu-BTC** (right).

Section 6: Vibrational studies and thermal activation process.

Section 7: FTIR spectra of **Eu-BTC** (a, b) and **Eu-BPDC** (c, d) under different thermal treatment (black, room temperature; blue, at 150 ºC; pink, at 200 ºC and red at 270 ºC).

Section 8: UV-vis profiles of the analytes and Eu-MOFs sensors.

Section 9: Stern-Volmer profile (R^2=0.9782) of the hypersensitive transition emission of **Tb-PSA** from 0 to 4000 ppm of $[UO_2]^{2+}$ (up). Same study in the 0-200 ppm (middle) and into the 400-4000 ppm of $[UO_2]^{2+}$(bottom).

Section 10: Stern-Volmer profile (R^2=0.9699) of the hypersensitive transition emission of **Tb-PSA** from 0 to 2340 ppm of Cu^{2+}(up). Same study in the 0-170 ppm (middle) and into the 340-2340 ppm of Cu^{2+} (bottom).

Section 11: Plot of N_2 sorption at 77.6 K versus pressure for **Eu-BTC'**.

Section 12: Sensor-analyte interaction studies.

Section 13: List of the contacts involving the guest residues and the **Eu-BPDC** host framework over the simulated dynamics.

Section 14: Portions of guest molecules in contact with the **Eu-BPDC** host framework.

Section 15: EDS profile for **MSM@Eu-BTC**.

Section 16: EDS profile for **IMZ@Eu-BTC**.

Section 17: EDS profile for **CLP@Eu-BTC**.
Section 18: EDS profile for **CLM@Eu-BTC**.
Section 19: EDS profile for **$[UO_2]^{2+}$@Eu-BTC**.
Section 20: EDS profile for **Cu^{2+}@Eu-BTC**.
Section 21: EDS profile for **TOL@Eu-BTC**.
Section 22: FTIR spectra of **CLM@Eu-BTC** (pink) in comparison with the corresponding of **Eu-BTC** (black) and **CLM** (red). The violet squares show the zones with remarkable band modifications.
Section 23: FTIR spectra of **CLM@Eu-BPDC** (pink) in comparison with the corresponding of **Eu-BPDC** (black) and **CLM** (red). The violet squares show the zones with remarkable band modifications.
Section 24: FTIR spectra of **MSM@Eu-BTC** (pink) in comparison with the corresponding of **Eu-BTC** (black) and **MSM** (red). The violet squares show the zones with remarkable band modifications.
Section 25: FTIR spectra of **MSM@Eu-BPDC** (pink) in comparison with the corresponding of **Eu-BPDC** (black) and **MSM** (blue). The violet squares show the zones with remarkable band modifications.
Section 26: FTIR spectra of **IMZ@Eu-BTC** (pink) in comparison with the corresponding of **Eu-BTC** (black) and **IMZ** (red). The violet squares show the zones with the most significant band modifications.
Section 27: FTIR spectra of **IMZ@Eu-BPDC** (pink) in comparison with the corresponding of **Eu-BPDC** (black) and **IMZ** (red). The violet squares show the zones with the most significant band modifications.
Section 28: FTIR spectra of **CLP@Eu-BTC** (pink) in comparison with the corresponding of **Eu-BTC** (black) and **CLP** (red). The violet squares show the zones with the most significant band modifications.
Section 29: FTIR spectra of **CLP@Eu-BPDC** (pink) in comparison with the corresponding of **Eu-BPDC** (black) and **CLP** (red). The violet squares show the zones with the most significant band modifications.

Section 1: Energy scheme[1] of the diverse electronic excited levels for trivalent lanthanides. The marked square denotes the hypersensitive transitions for Eu^{3+} and Tb^{3+}.

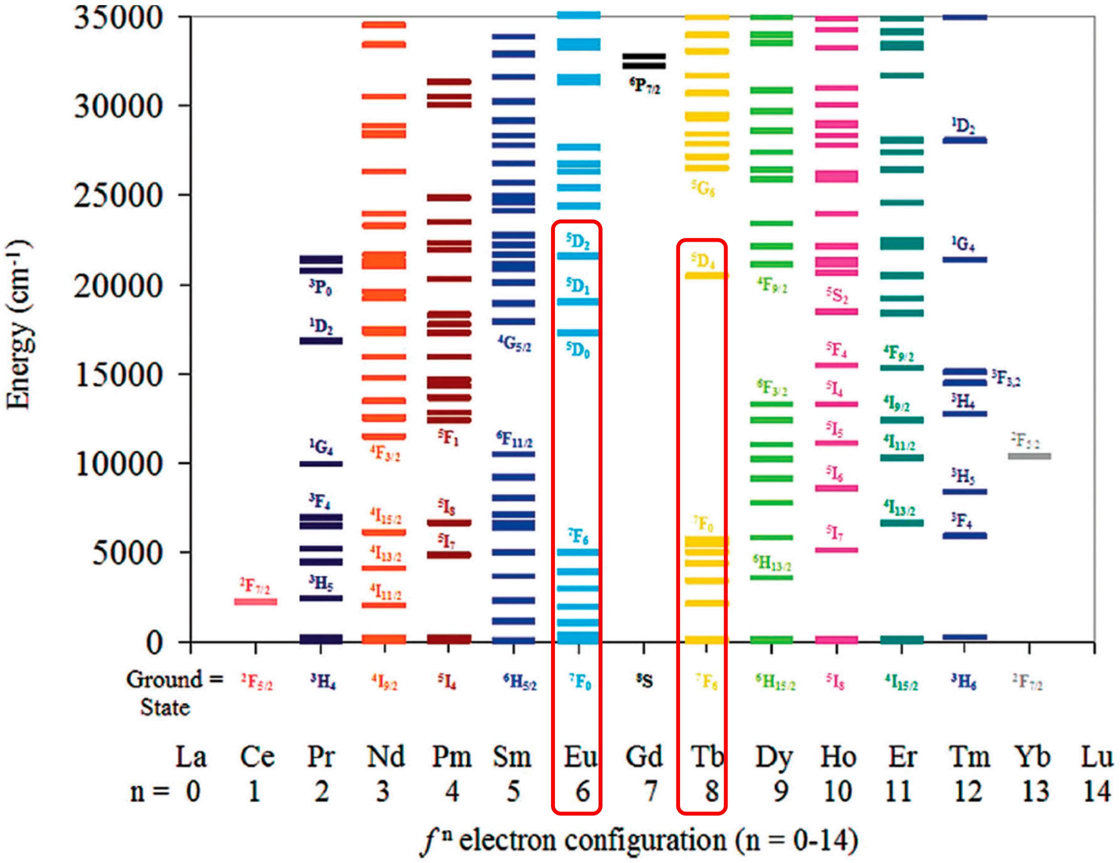

Section 2: Synthesis procedures.

The sensor materials were obtained as crystalline solids under solvothermal conditions using 43 or 120 mL Teflon-lined Parr reactors. The solid reactants and solvents were used as received without purification from Sigma-Aldrich [4,4'-bipyridine, $Eu(NO_3)_3{\cdot}6H_2O$, $Tb(NO3)_3{\cdot}5H_2O$, $UO_2(NO_3)_2{\cdot}6H_2O$, $CuCl_2$, $MnCl_2.6H_2O$, toluene, N,N'-dimethylformamide (DMF), 1,3,5-benzenetricarboxylic acid (H_3BTC, $H_6C_9O_6$), 4,4'-biphenyldicarboxylic acid (H_2BPDC, $H_{10}C_{14}O_4$), 2-phenylsuccinate acid (H_2PSA, $H_{10}C_{10}O_4$), thriethylamine (TEA)] and Merck (Ethanol). The agrochemicals were used as received from Sigma-Aldrich, without further purification.

$[Eu_2(C_{10}H_8O_4)_3(H_2O)]$ (**Eu-PSA** or **Tb-PSA**). The crystalline powders were obtained under solvothermal synthesis according with the procedure previously reported (Reference 28 in main manuscript): shortly, both compounds were synthesized by dissolving 1 mmol of H_2PSA (0.194 g) and the lanthanide salts (chlorides or nitrates, 1 mmol) in a mixture of 30 mL of distilled water and 30 mL of EtOH adjusting the pH to 4.5 with 1 mmol of 4,4'-bipyridine. The mixtures were heated at 160 ºC for 72 h.

$[Eu_2(C_{14}H_8O_4)_3(H_2O)_2]$.DMF (**Eu-BPDC**). The compound was obtained according to the procedure previously reported with minimal modifications (Reference 29 in main manuscript): 0.1 mmol of $Eu(NO_3)_3{\cdot}5H_2O$ (0.043 g), 0.05 mmol of

[1] Y. Cui, Y. Yue, G. Qian, B. Chen, *Chem. Soc. Rev.*, **2012**, 112(2), 1126-1162.

H_2BPDC (0.012 g) were dissolved in a mixture of 10 mL of DMF and 2 mL of EtOH. One drop of TEA was added and left undisturbed at 60 ºC for 8 days instead of 15 days, giving rise to needle-shaped colorless crystals.

$[Eu_2(C_9H_3O_4)_3(H_2O)]\cdot DMF$ (**Eu-BTC**). The compound was solvothermally obtained by mixing 0.33 mmol of $Eu(NO_3)_3\cdot 5H_2O$ (0.141 g) and 0.33 mmol of H_3BTC (0.07 g) in 10 mL of DMF at 85 ºC for 6 days (Reference 30 in main manuscript).

After cooling the mixture, all the crystalline products were washed with water/ethanol mixtures.

Afterwards, the powder samples were characterized by powder X-ray diffraction (PXRD), with the purpose of verifying the presence of the required MOF phase, by comparison of the experimental and simulated PXRD profile (see Supp. Mater. section 3).

Section 3: PXRD patterns of **Eu-BPDC**, **Eu-BTC**, **Tb-PSA** and **Eu-PSA** in comparison with the corresponding simulated diffractograms.

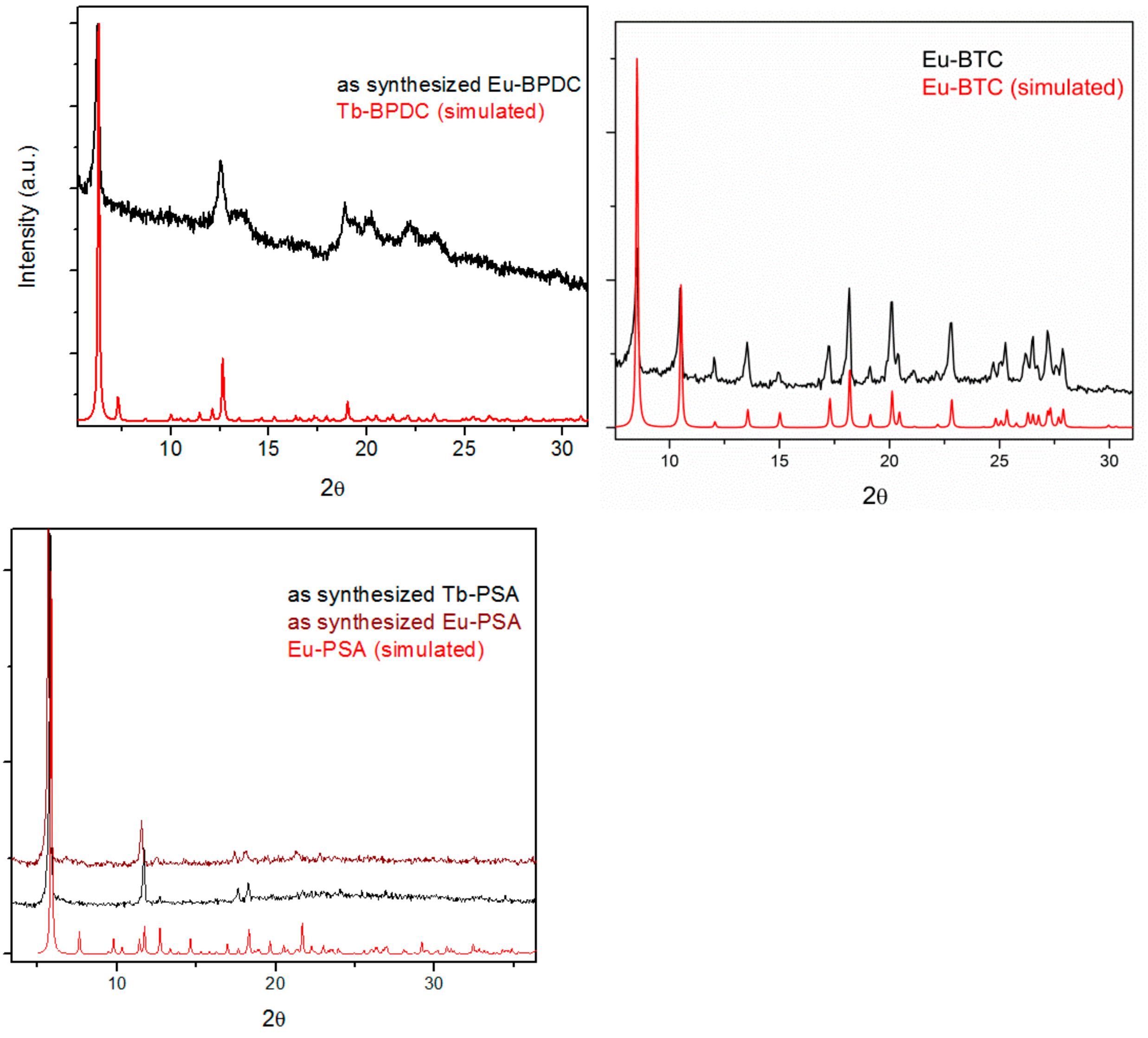

Section 4: PXRD patterns of **Eu-BPDC**, **Eu-BTC**, **Tb-PSA** and **Eu-PSA** after exposition to uranyl cation.

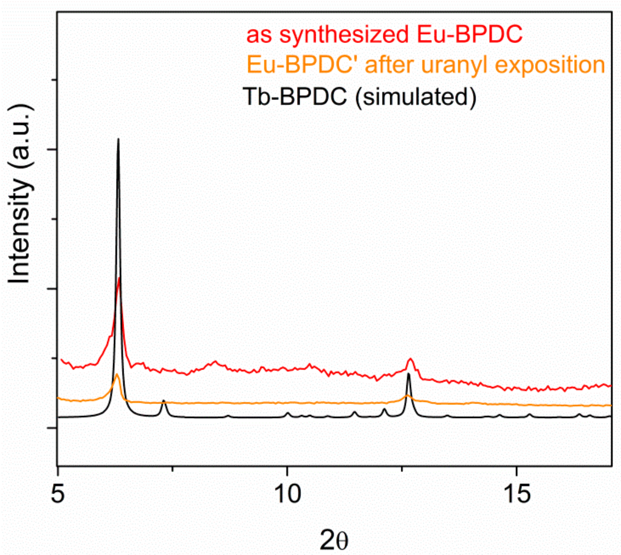

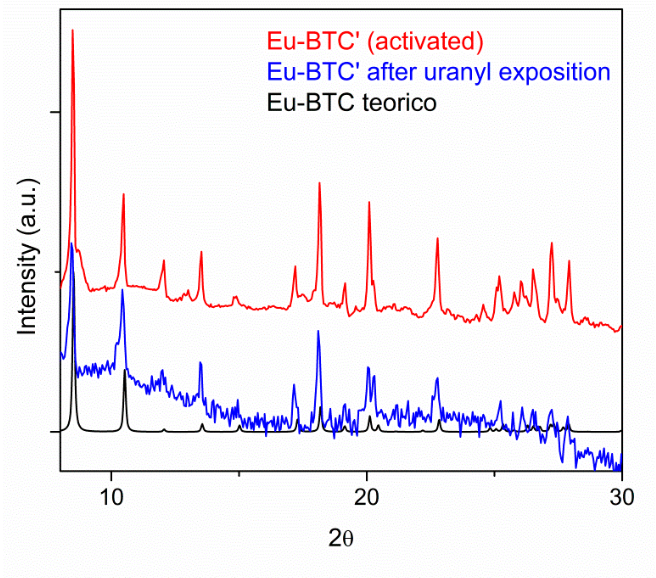

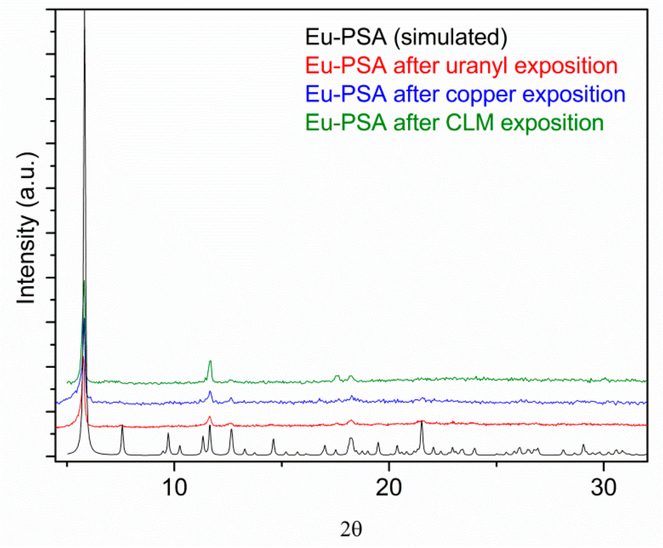

Section 5: TGA/DTA curves for **Eu-BPDC** (left) and **Eu-BTC** (right).

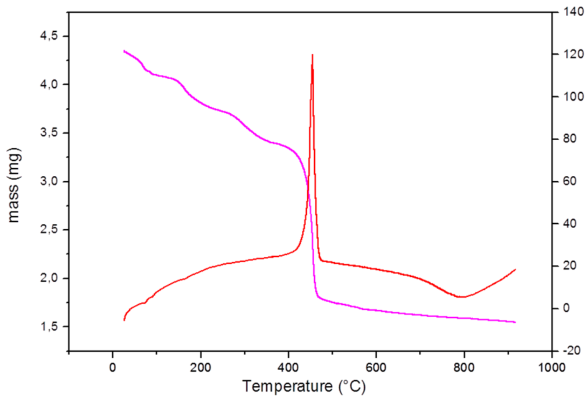

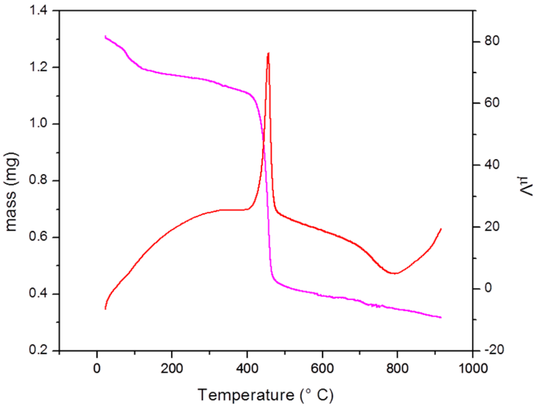

Section 6: Vibrational studies and thermal activation process.

Trough vibrational characterization, it was possible to identify bands related to the asymmetric and symmetric modes of carboxylate groups (1581-1412 cm^{-1} in **Eu-BPDC** and 1566-1381 cm^{-1} for **Eu-BTC**), coordinated water (3417 cm^{-1} for **Eu-BPDC** and 3410 cm^{-1} for **Eu-BTC**) and guest DMF molecules (2923, 1651 cm^{-1} for **Eu-BPDC** and 3093-2862, 1658 cm^{-1} for **Eu-BTC**). When temperature increased (up to 270 ºC), DMF vibrational modes completely disappeared, according with the procedure made with the analogous **Dy-BTC**.[2] There are not reported activation procedures neither for **Eu-BPDC** nor for other isostructural Ln-BPDC phases. Nevertheless, based on our thermal studies it was possible to conclude that the heating process of the sample until 270 °C, should efficiently evacuate guest DMF molecules preserving the structure without detectable phase transformations (Supp. Mater. section 5).

Section 7: FTIR spectra of **Eu-BTC** (a, b) and **Eu-BPDC** (c, d) under different thermal treatment (black, room temperature; blue, at 150 ºC; pink, at 200 ºC and red at 270 ºC).

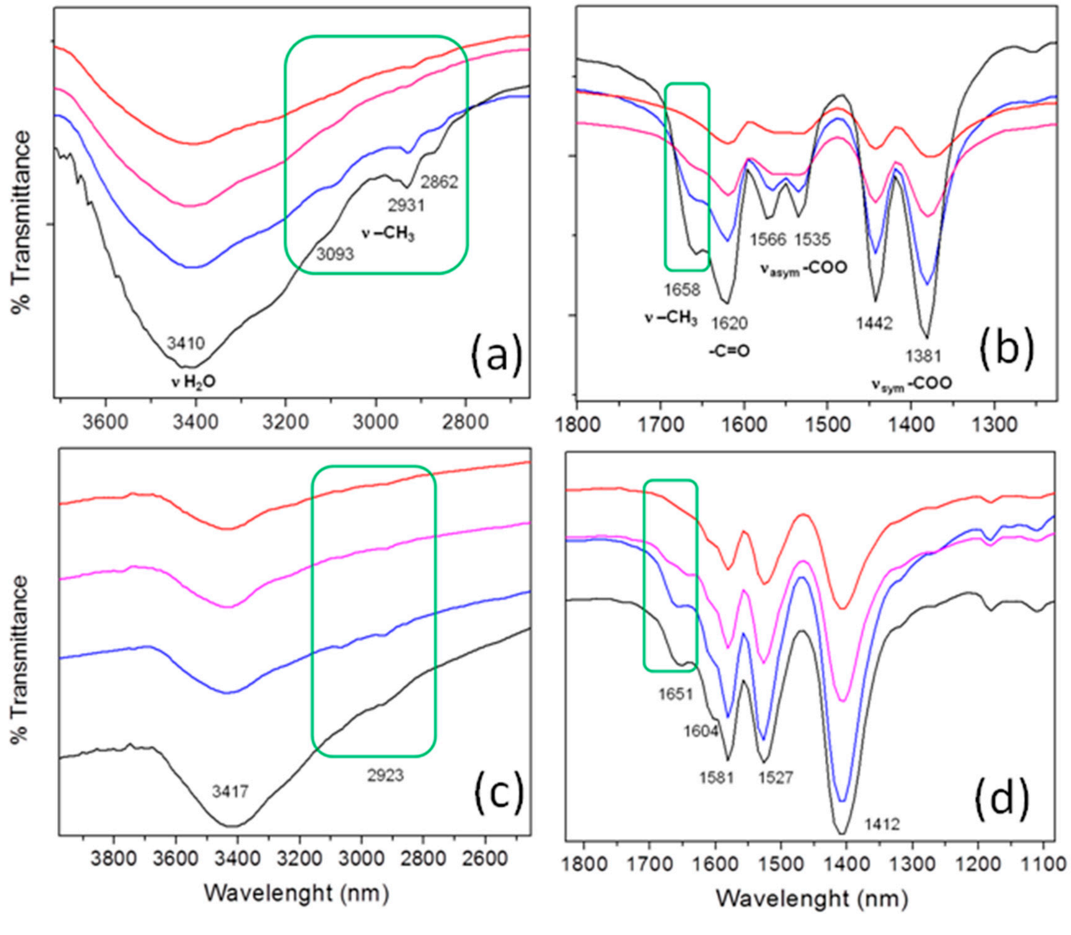

Section 8: UV-vis profiles of the analytes and Eu-MOFs sensors.

---

[2] X. Guo, G. Zhu, Z. Li, F. Sun, Z. Yang, S. Qiu., *ChemComm.*, **2006**, 3172-3174.

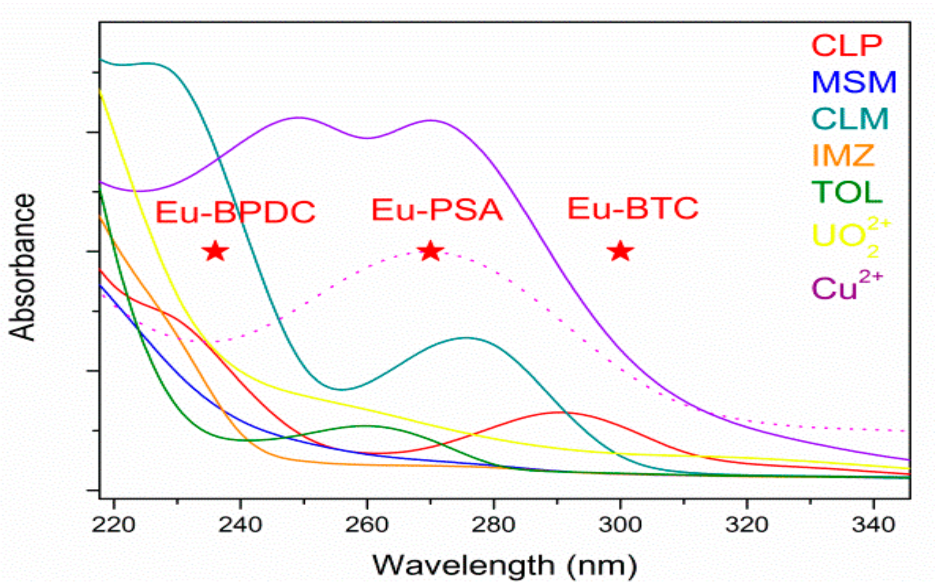

Section 9: Stern-Volmer profile (R^2=0.9782) of the hypersensitive transition emission of **Tb-PSA** from 0 to 4000 ppm of $[UO_2]^{2+}$ (up). Same study in the 0-200 ppm (middle) and into the 400-4000 ppm of $[UO_2]^{2+}$(bottom).

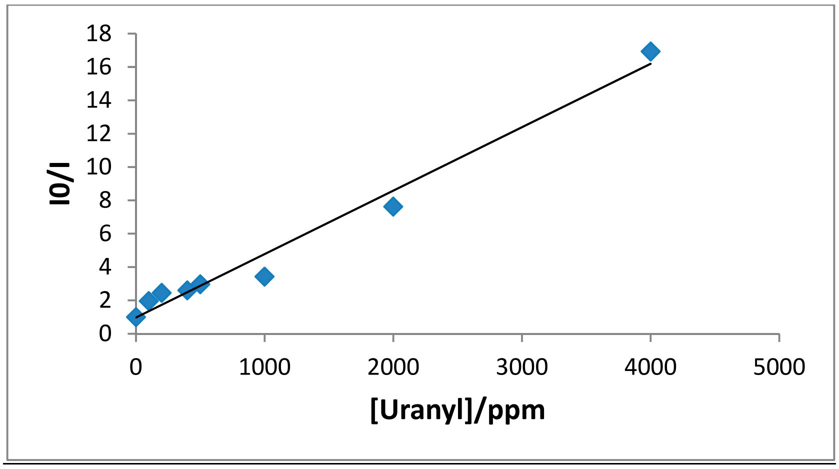

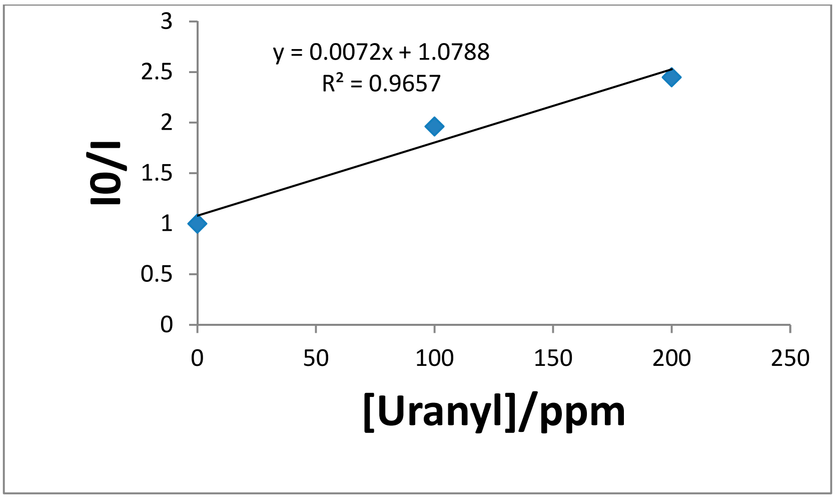

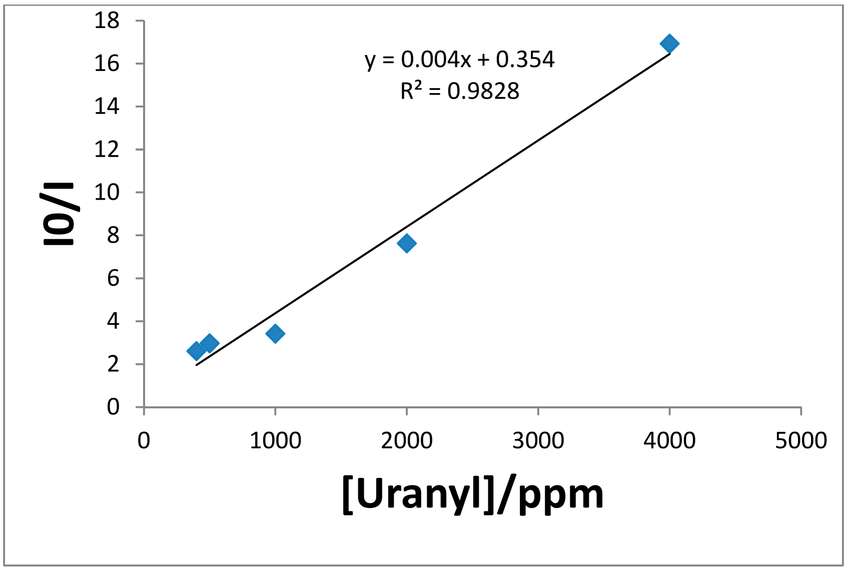

Section 10: Stern-Volmer profile (R^2=0.9699) of the hypersensitive transition emission of **Tb-PSA** from 0 to 2340 ppm of Cu^{2+}(up). Same study in the 0-170 ppm (middle) and into the 340-2340 ppm of Cu^{2+} (bottom).

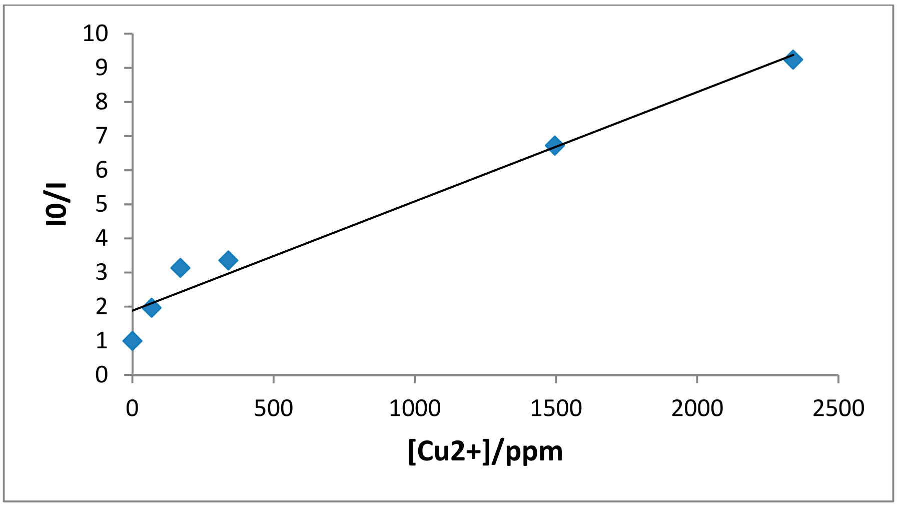

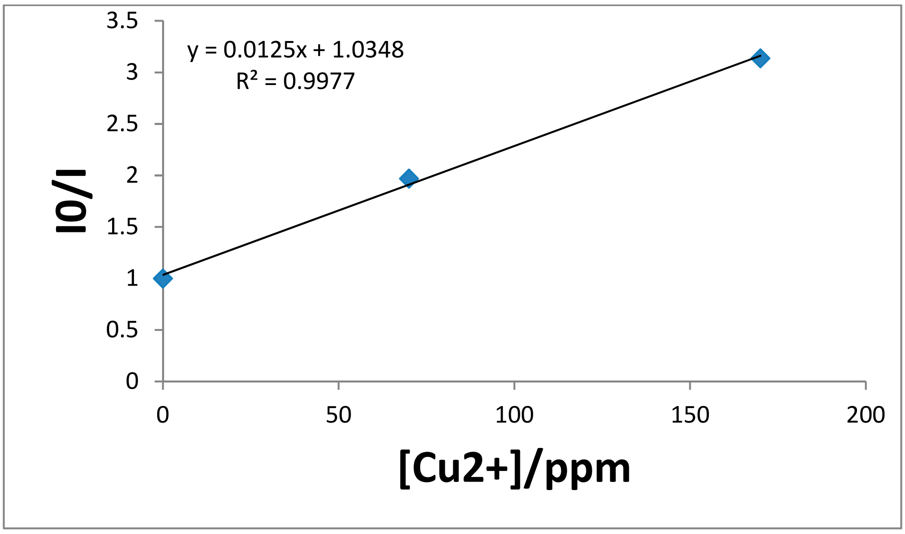

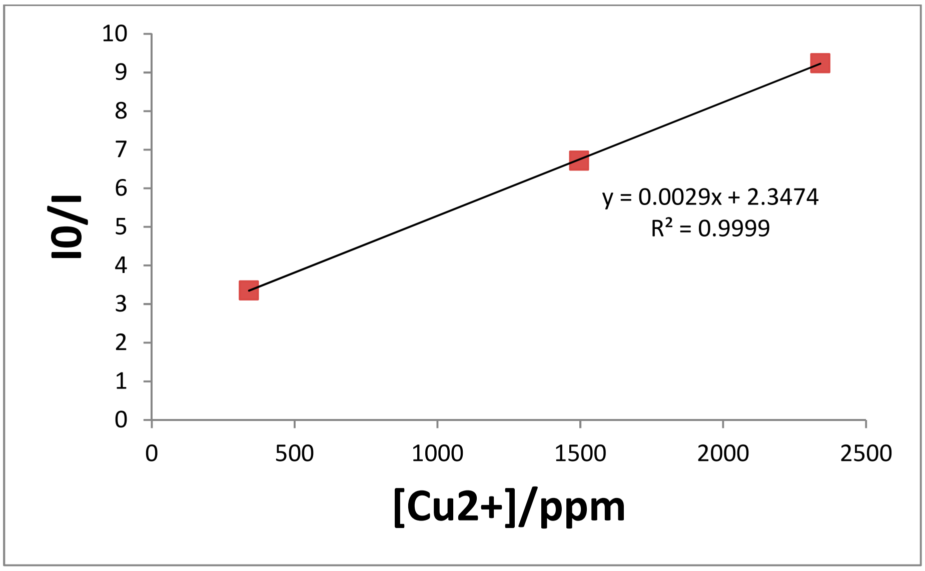

Section 11: Plot of N_2 sorption at 77.6 K versus pressure for **Eu-BTC'**.

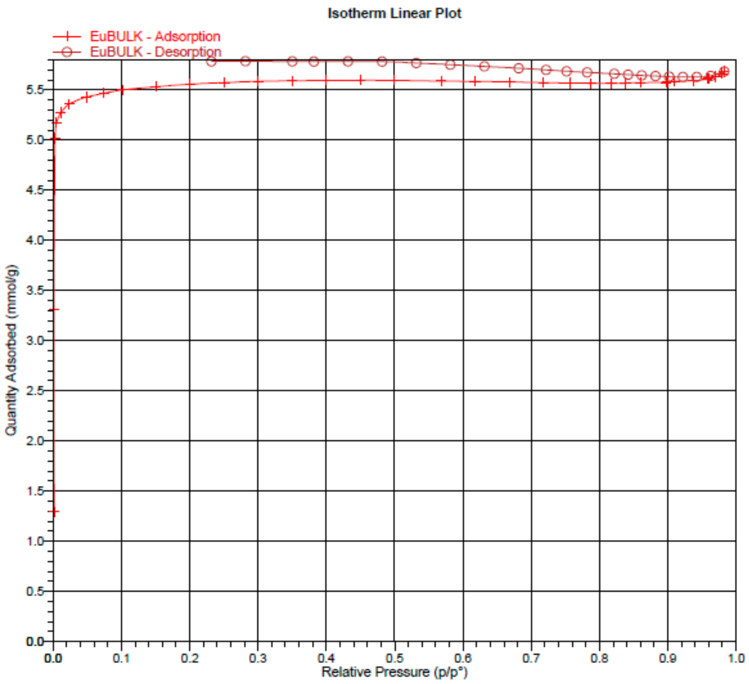

Section 12: Sensor-analyte interaction studies

To further characterize the intermolecular interactions between the host framework (**Eu-BPDC** taken as model) and the guest molecules, we examined the following two structural parameters from the structural assemble of MD trajectory: 1) the mass-weighted RMSD of the guest inside a pore of the host framework (this could help us to evaluate how much the guest molecule moves inside a pore of the host framework); 2) the fractional contacts between Eu atoms of the host framework and the heavy atoms X (X=O, N, or S) of the guest molecules in the structure produced by MD simulation.

The mass-weighted RMSD of the guest inside a pore of the framework allowed us to analyze the dynamic flexibility and the conformational sampling of the guest molecule. The mass-weighted root-mean-square deviations (rmsd's) between snapshots obtained during the course of the trajectory and the original starting coordinates were calculated and plotted for each guest component (i.e., **CLM**, **CLP**, **IMZ** and **MSM**, Figures 14 in main manuscript). As show in Supp. Mater. Appendix 1, all guests reach a plateau in a short simulation time. Figure 14 and the Table in Supp. Mater. section 13 show that the guests molecules displays no significant structural deviation from its starting coordinates RMSD ≤ 1.23 Å, ≤ 1.58 Å, ≤ 1.26 Å, ≤ 2.63 Å for **CLM**, **CLP**, **IMZ** and **MSM**, respectively. Moreover, these results indicate that the guests flexibilities changes only to a limited extent. From the RMSD results, we assume a well-behaved guest-host model system that achieves a reasonable amount of sampling for each guest molecules.

The Table of Supp. Mater. section 13 lists the contacts involving the guest residues and the host framework over the simulated dynamics. We found that at 298 K during 100 ns of simulation time, the **CLM** molecule contacts the host framework by the sulfamoyl moiety (O4 and O5) as well as by the carbamoyl-amino moiety (O7) and the carbonyl group of the ethyl-benzoate moiety (O6). Similar modes of contact are found in the case of the **MSM** molecule, which contacts the host framework by the sulfamoyl moiety (O5) as well as by the carbamoyl-amino moiety (O6) and the carbonyl group of the methyl-benzoate moiety (O3). The above Eu-O interatomic contacts were found between 3.96-2.57 and 3.93-2.64 Å for the **CLM** or **MSM** molecules, respectively. The portions of guest molecules in contact with the **Eu-BPDC** host framework are displayed in Supp. Mater. section 14.

Experimental EDS and FTIR results support the majority of the contacts identified in our model system. In Supp. Mater. sections 15-21 it is clear the adsorption of toxics by the presence of specific peaks in the EDS profiles of **X@Eu-BTC**. In addition, in the case of the FTIR spectrum of **CLM@Eu-BTC** (Supp. Mater. section 22), the presence of the carbonyl band (1720 cm^{-1}) and the symmetric and asymmetric stretching modes (1327, 1280 and 1165, 1118 cm^{-1}) of the sulfone group allowed us to say that **CLM** compound was adsorbed to the sensor. For the case of **CLM@Eu-BPDC** (Supp. Mater. section 23) a shift of 29 $cm^{-}1$ respect to the free carbonyl group of free **CLM** (1720.2 cm^{-1}) could be seen, attributable to the coordination to Eu ions. On the other hand, for both cases the depletion of the stretching carbonyl mode of DMF (1658 cm^{-1}), would indicate a complete evacuation during the ultrasonication in methanol medium. When spectra of **Eu-BTC** and **MSM@Eu-BTC** (Supp. Mater. section 24) were compared, some new bands appeared in the latter, related with carbonyl of ester function and the secondary amide group of free MSM is shifted ($\Delta\nu$~8 cm^{-1}) in **MSM@Eu-BTC** at 1735 cm^{-1}. It would suggest that **MSM** molecules interact with the metallic centers via carbonyl coordination. The symmetric stretching of the aromatic ester portion, located at 1126 cm^{-1} in MSM shifted to 1118 cm^{-1} while the asymmetric one at 1257 cm^{-1} remained the same in both spectra. The band located at 1296 cm^{-1} related with sulfone $–SO_2$ group stretching changed neither in intensity nor in position which indicated a poor interaction of Eu centers with this part of the molecule. Focusing on **MSM@Eu-BPDC** (Supp. Mater. section 25), a shift of 2 cm^{-1} is seen in the $–SO_2$ group stretching due an interaction with the lanthanide centers (1057 *vs* 1059 cm^{-1}).

A complete agreement between our model system and the FTIR results is also found for the **IMZ** compound. Table in Supp. Mater. section 10 shows that the imidazole moiety (N5) of **IMZ** contacts the host framework at an equilibrium

distance of ~ 2.70 Å. Analyzing the spectrum of **IMZ@Eu-BTC** (Supp. Mater. section 26), it was noticeable an increment of the bands located at 2931 and 2854 cm^{-1} related to the stretching modes of $–CH_2$ group of **IMZ** molecules. By following the band located at 1658 cm-1 in the **IMZ@Eu-BTC** system, it was possible to confirm that DMF completely disappeared under ultrasonication removal. For the case of **IMZ@Eu-BPDC**, a deeper interaction could be noticeable due the shift ($\Delta\nu$=6.6 cm^{-1}) in the nitrogen imidazole group indicating a ν(Eu-N) interaction (located band at 252.6 cm^{-1}) (Supp. Mater. section 27).

Respect to **CLP@Eu-BPDC**, a new band appeared at 229.5 cm^{-1} ascribed to Eu-N interaction, nevertheless this band is weak and low intense suggesting a poor interaction. There is no evidence in vibrational study of carbonyl interaction with europium centers (Supp. Mater. section 28).

For the **CLP** molecule, our model system shows significant contact between the host framework and the sulfur atom of the phosphorothioate moiety (S4) (Q≈100%, ~2.87 Å). In addition, our model exhibits few long-range contacts (Q≈0.5%, ~3.96 Å) between the host framework and the oxygen atom of the O-diethyl moieties of **CLP**. Nevertheless, these Eu-S and Eu-O contacts are not detected from the FTIR results; however, the vibrational studies shows a Eu-N contact for the pirydin moiety of CLP at ν(Eu-N) 229.5 cm^{-1}. This contact is found in our model system for a Q≈0.6% at ~3.83 Å.

Section 13: List of the contacts involving the guest residues and the **Eu-BPDC** host framework over the simulated dynamics.

	Contact	N°frames	Frac.%	Avg(SD)
CLM				
	O4	20000	100.0	2.57(0.07)
	O6	20000	100.0	3.03(0.15)
	O7	12446	62.2	3.82(0.11)
	O5	3	0.1	3.96(0.02)
CLP				
	S4	20000	100.0	2.87(0.07)
	N9	117	0.6	3.83(0.11)
	O7	91	0.5	3.96(0.04)
IMZ				
	N5	20000	100.0	2.70(0.07)
MSM				
	O5	20000	100.0	2.64(0.09)
	O3	17774	88.9	2.74(0.11)
	O6	2169	10.8	3.91(0.07)
	O2	53	0.3	3.93(0.06)

Section 14: Portions of guest molecules in contact with the **Eu-BPDC** host framework.

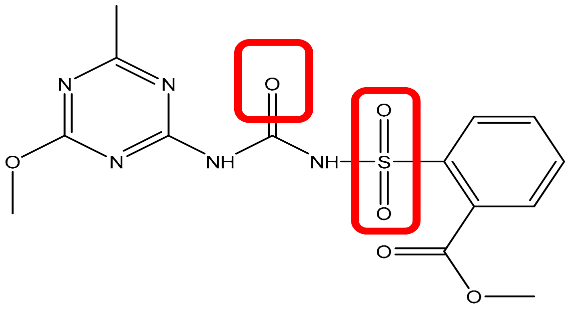

MSM

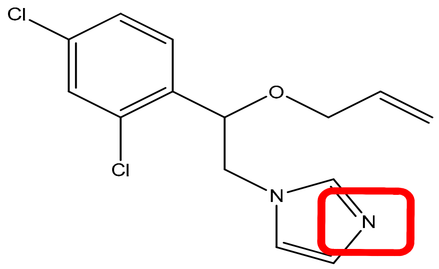

IMZ

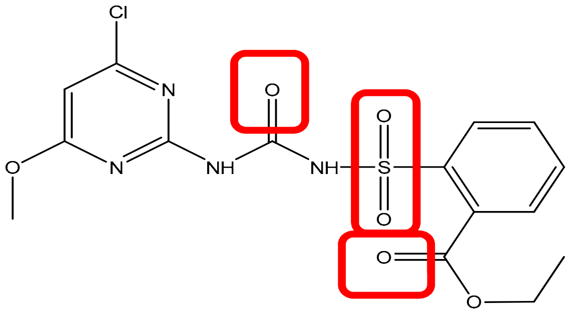

CLM

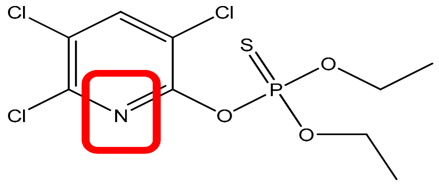

CLP

Section 15: EDS profile for **MSM@Eu-BTC**.

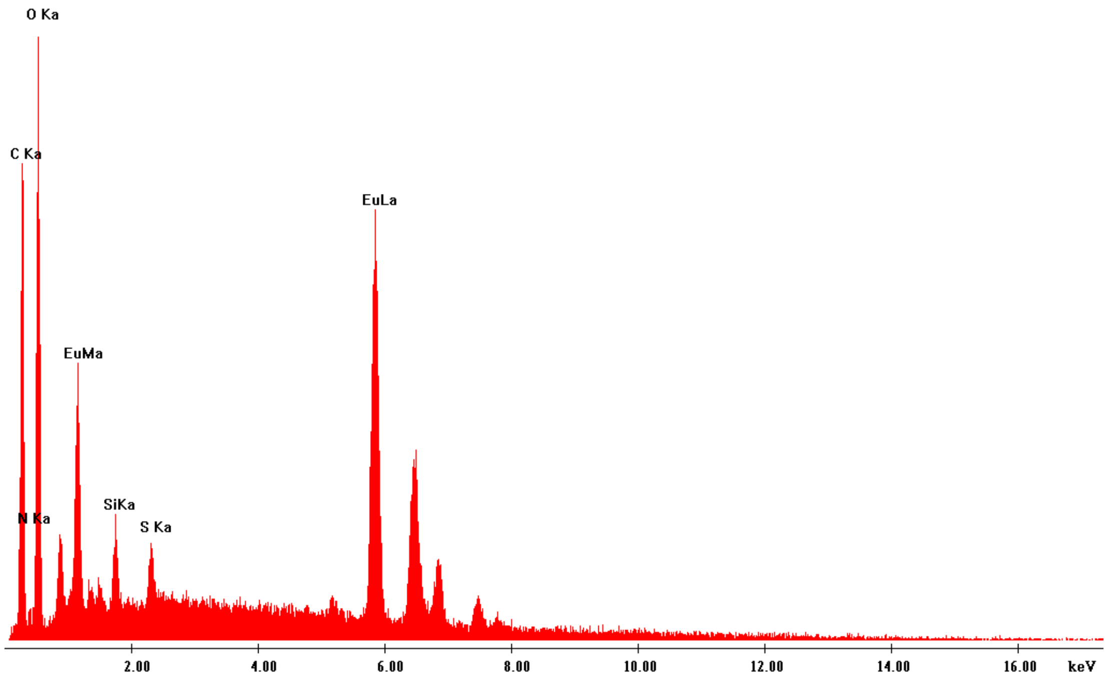

Section 16: EDS profile for **IMZ@Eu-BTC**.

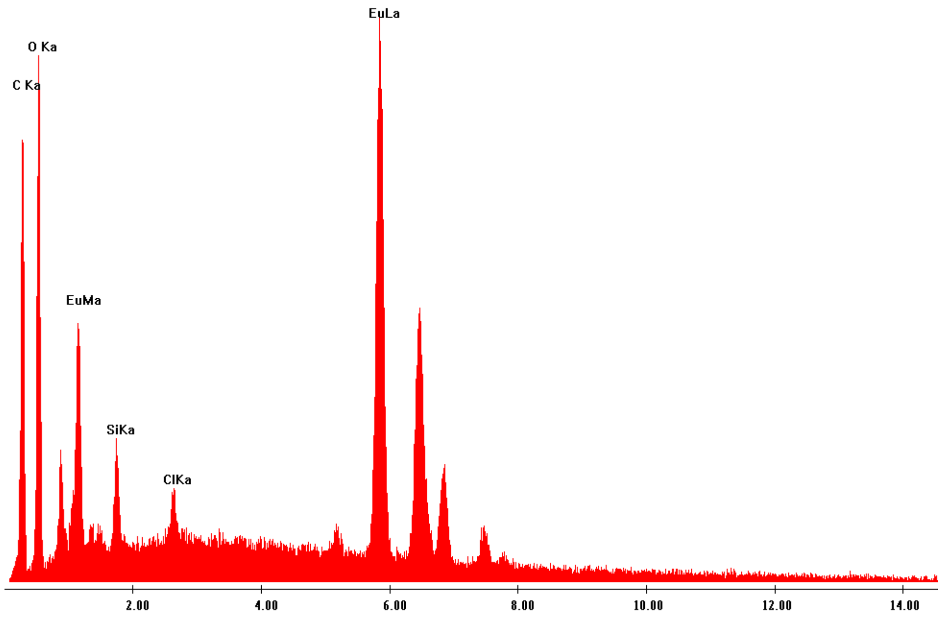

Section 17: EDS profile for **CLP@Eu-BTC**.

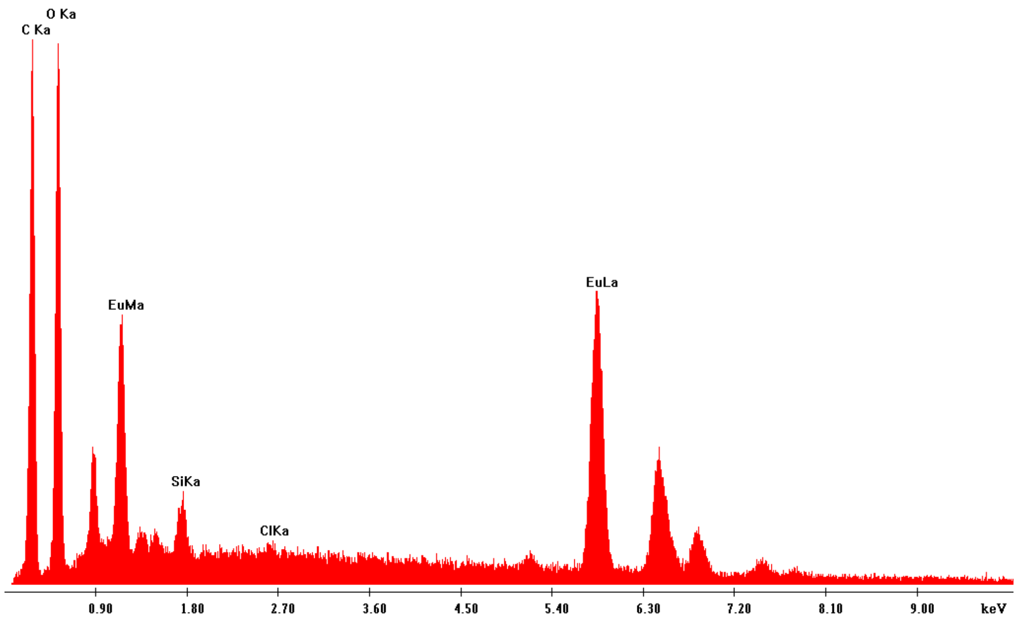

Section 18: EDS profile for **CLM@Eu-BTC**.

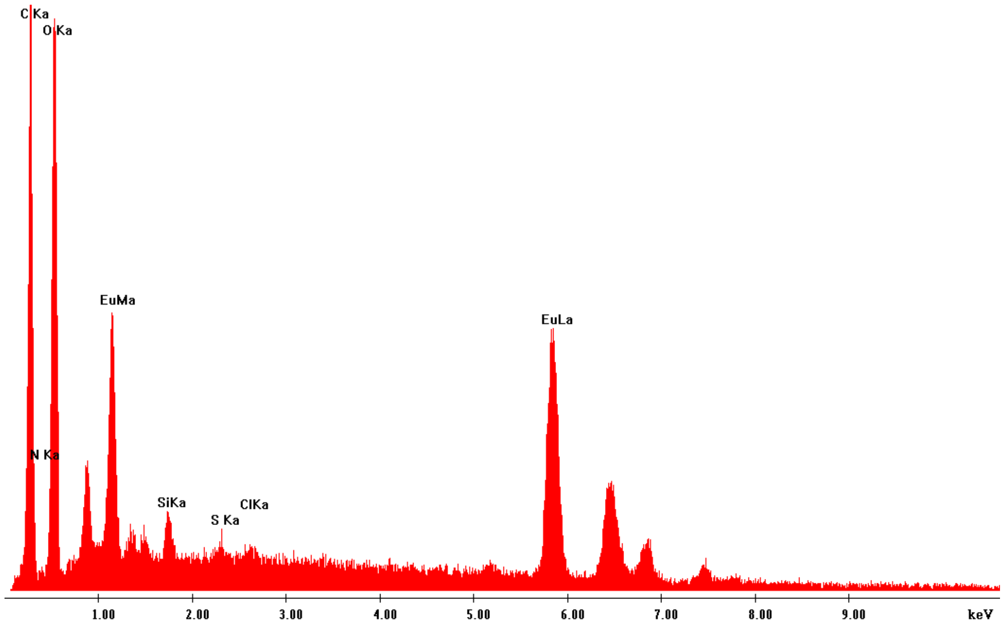

Section 19: EDS profile for $\mathbf{[UO_2]^{2+}}$**@Eu-BTC**.

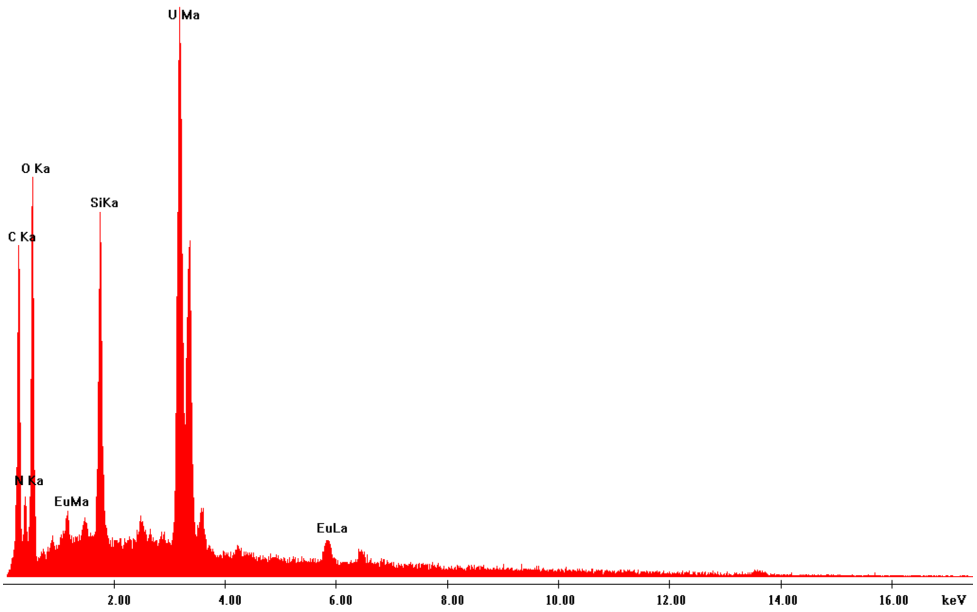

Section 20: EDS profile for $\mathbf{Cu^{2+}}$**@Eu-BTC**.

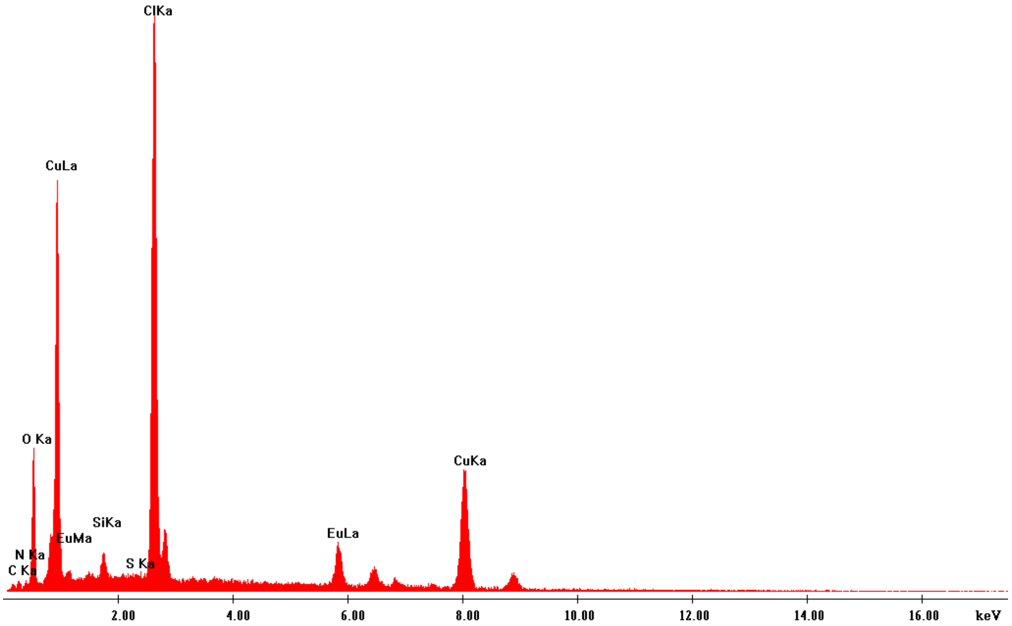

Section 21: EDS profile for **TOL@Eu-BTC**.

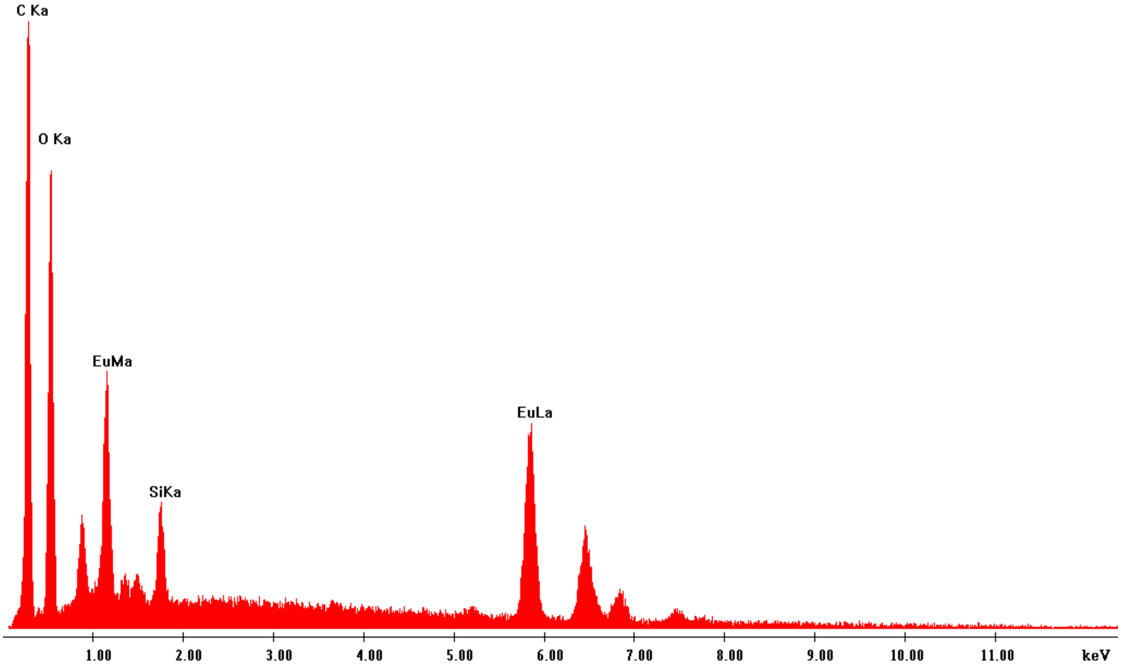

Section 22: FTIR spectra of **CLM@Eu-BTC** (pink) in comparison with the corresponding of **Eu-BTC** (black) and **CLM** (red). The violet squares show the zones with remarkable band modifications.

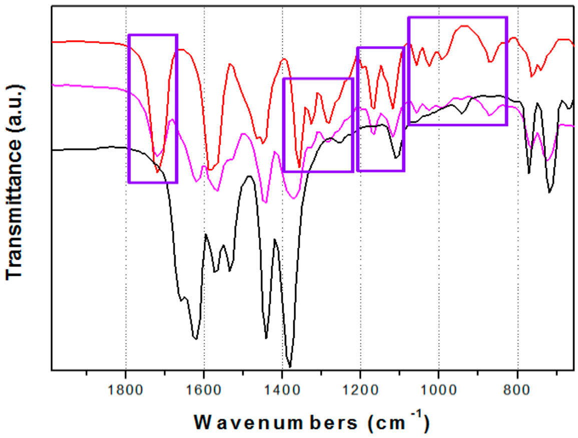

Section 23: FTIR spectra of **CLM@Eu-BPDC** (pink) in comparison with the corresponding of **Eu-BPDC** (black) and **CLM** (red). The violet squares show the zones with remarkable band modifications.

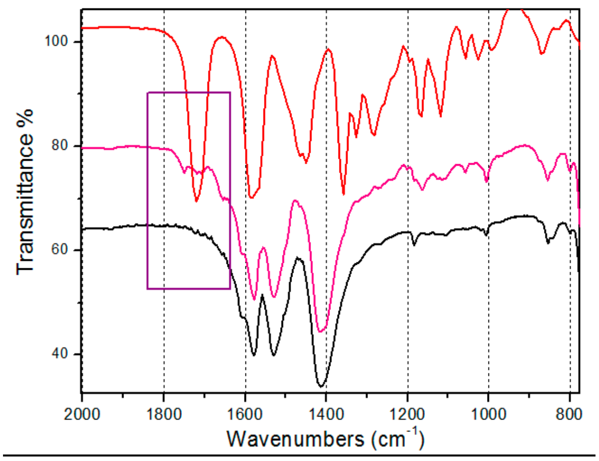

Section 24: FTIR spectra of **MSM@Eu-BTC** (pink) in comparison with the corresponding of **Eu-BTC** (black) and **MSM** (red). The violet squares show the zones with remarkable band modifications.

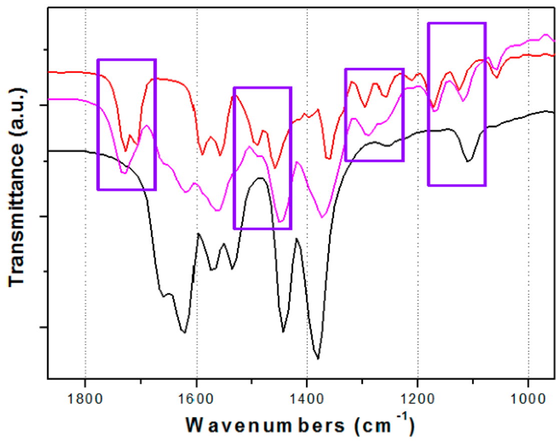

Section 25: FTIR spectra of **MSM@Eu-BPDC** (pink) in comparison with the corresponding of **Eu-BPDC** (black) and **MSM** (blue). The violet squares show the zones with remarkable band modifications.

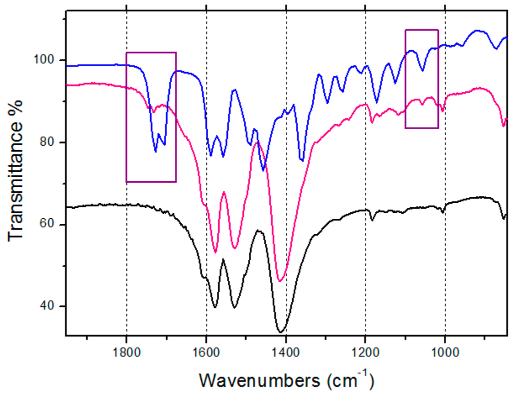

Section 26: FTIR spectra of **IMZ@Eu-BTC** (pink) in comparison with the corresponding of **Eu-BTC** (black) and **IMZ** (red). The violet squares show the zones with the most significant band modifications.

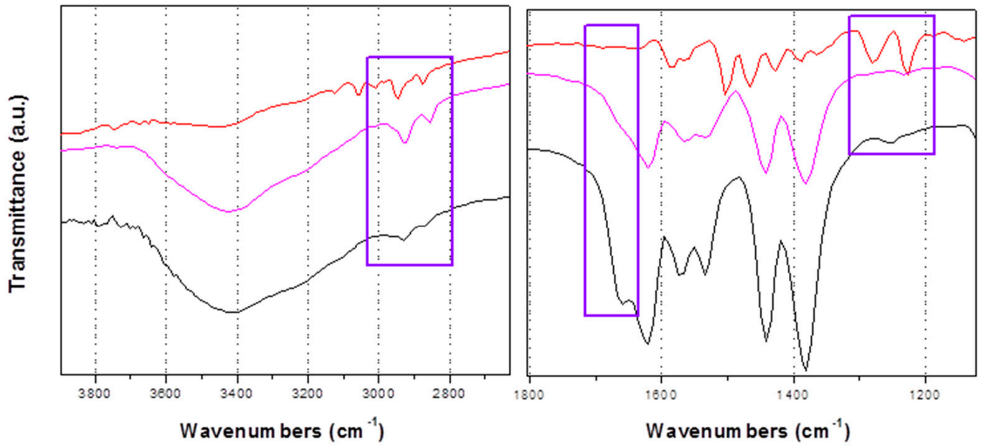

Section 27: FTIR spectra of **IMZ@Eu-BPDC** (pink) in comparison with the corresponding of **Eu-BPDC** (black) and **IMZ** (red). The violet squares show the zones with the most significant band modifications.

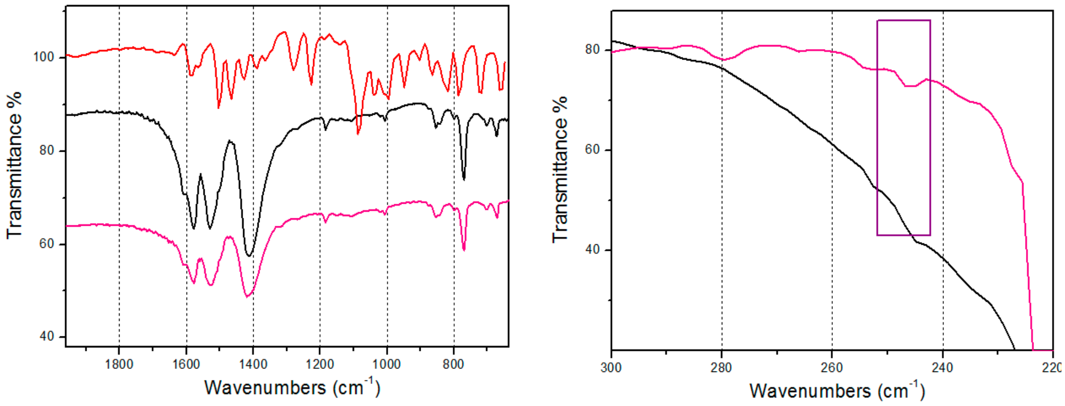

Section 28: FTIR spectra of **CLP@Eu-BTC** (pink) in comparison with the corresponding of **Eu-BTC** (black) and **CLP** (red). The violet squares show the zones with the most significant band modifications.

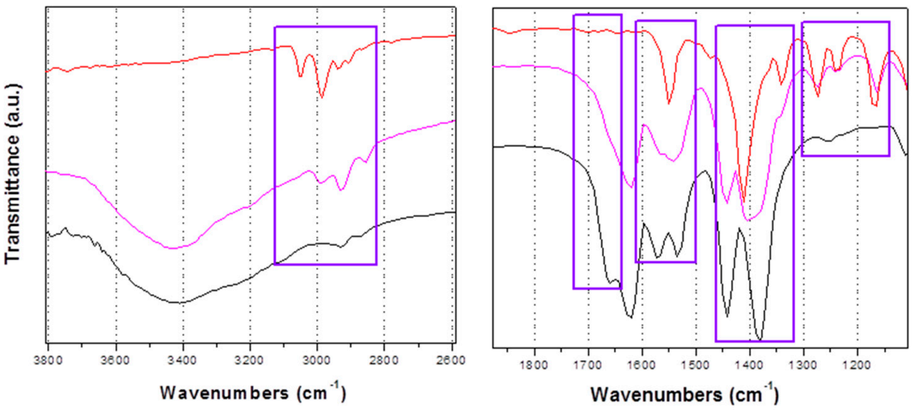

Section 29: FTIR spectra of **CLP@Eu-BPDC** (pink) in comparison with the corresponding of **Eu-BPDC** (black) and **CLP** (red). The violet squares show the zones with the most significant band modifications.

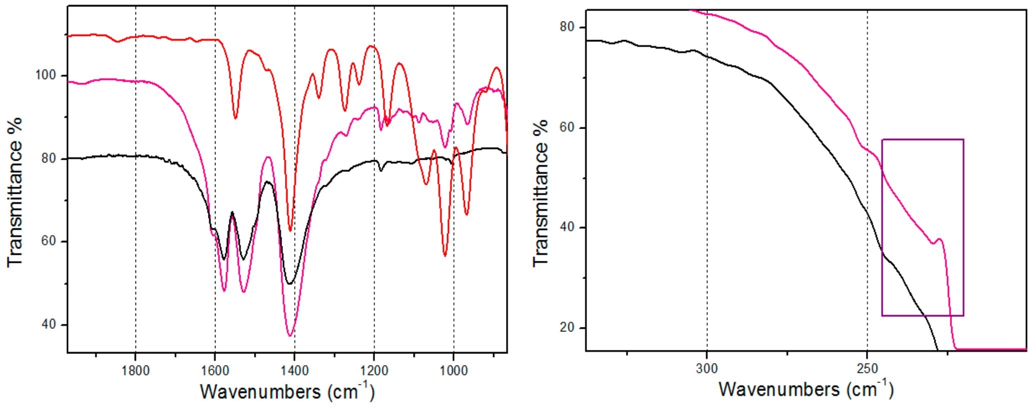

Supplement: Supplementary file 1 [file sensors-19-01260-s001.pdf]
